# Multivariate Meta-Analysis of Genetic Association Studies: A Simulation Study

**DOI:** 10.1371/journal.pone.0133243

**Published:** 2015-07-21

**Authors:** Binod Neupane, Joseph Beyene

**Affiliations:** 1 Department of Clinical Epidemiology and Biostatistics, McMaster University, Hamilton, Ontario, Canada; 2 Department of Mathematics and Statistics, McMaster University, Hamilton, Ontario, Canada; Johns Hopkins Bloomberg School of Public Health, UNITED STATES

## Abstract

In a meta-analysis with multiple end points of interests that are correlated between or within studies, multivariate approach to meta-analysis has a potential to produce more precise estimates of effects by exploiting the correlation structure between end points. However, under random-effects assumption the multivariate estimation is more complex (as it involves estimation of more parameters simultaneously) than univariate estimation, and sometimes can produce unrealistic parameter estimates. Usefulness of multivariate approach to meta-analysis of the effects of a genetic variant on two or more correlated traits is not well understood in the area of genetic association studies. In such studies, genetic variants are expected to roughly maintain Hardy-Weinberg equilibrium within studies, and also their effects on complex traits are generally very small to modest and could be heterogeneous across studies for genuine reasons. We carried out extensive simulation to explore the comparative performance of multivariate approach with most commonly used univariate inverse-variance weighted approach under random-effects assumption in various realistic meta-analytic scenarios of genetic association studies of correlated end points. We evaluated the performance with respect to relative mean bias percentage, and root mean square error (RMSE) of the estimate and coverage probability of corresponding 95% confidence interval of the effect for each end point. Our simulation results suggest that multivariate approach performs similarly or better than univariate method when correlations between end points within or between studies are at least moderate and between-study variation is similar or larger than average within-study variation for meta-analyses of 10 or more genetic studies. Multivariate approach produces estimates with smaller bias and RMSE especially for the end point that has randomly or informatively missing summary data in some individual studies, when the missing data in the endpoint are imputed with null effects and quite large variance.

## Introduction

In genetic association studies of complex traits, estimation of the average effects of genetic variants on one or multiple quantitative phenotypic traits such as systolic blood pressure (SBP), diastolic blood pressure (DBP), blood triglycerides level (TG), low density lipoprotein (LDL) and high density lipoprotein (HDL) levels, etc. could be of interest. If two or more of these traits are measured in the same set of individuals, they may be correlated as they could be simultaneously influenced by the same gene(s) (pleiotropic effects) and/or environment (e.g., high dietary fat intake) in the same individuals [1,2]. Hence the true risks (e.g., log-odds ratios per one copy increase in the number of mutant/minor allele in a genotype at a DNA locus) of a causal gene on such correlated traits may be correlated across studies and corresponding estimates of risks may be correlated within studies. In individual studies, if risks estimates of different groups are obtained compared to a common referent group, then the estimates could be correlated within studies. For example, in genetic association studies the estimates of two log-odds ratios measuring the risks of a disease or phenotype in two groups carrying one and two copies of mutant risk allele as compared to a group carrying none are correlated within a study.

Multivariate approach could be used to jointly synthesize such correlated end points. (An 'endpoint' in the context of meta-analysis is an effect parameter to be estimated). It can exploit the between and/or within-study correlation structure to yield more efficient or precise estimates while univariate approach ignores such correlation structure [3,4]. It has been analytically shown to produce similar or more précised pooled estimates for correlated endpoints [5]. Also, simulation studies in clinical studies settings have shown that it can performs superior particularly for the endpoint with randomly or informatively missing study-wise summary data [3,6,7]

However, there are some practical issues with the use of multivariate approach in meta-analysis. First, for a small meta-analysis or for situation where between-study variation is relatively small compared to within-study variation, the multivariate method often estimates the between-study correlation at the boundary of parameter space (−1 or +1) [6,8]. This is thought to result in upwardly biased estimates of between-study variances and consequently imprecise pooled estimates [6]. Next, when the dimension, *p*, of multiple endpoints increases, estimation problem under multivariate random-effect meta-analysis becomes more complex because the effective number of parameters to be estimated is *p*(*p* + 1)/2. For example, when *p = 3*, a 3-variate meta-analysis requires the estimation of a total of six between study variances and correlation parameters simultaneously while a univariate meta-analysis requires estimation of just one between-study variance parameter at a time. Therefore, even when the end points are highly correlated, the use of multivariate approach can be prohibitive or may offer no clear advantage especially when number of studies is small or between-study variances are smaller compared to within-study variances. Despite advantages in theory, recent studies summarizing the empirical meta-analysis studies found that the improvement on the bias or precision of the pooled estimates is not remarkable from multivariate analysis compared to univariate in most applications [9,10,11]. Finally, univariate analysis is simpler and easier to understand and conduct than multivariate approach [4].

Given the above-discussed promises and issues of multivariate meta-analysis, it is not clear when its application may be preferable (i.e., whether it offers any practical advantage) to univariate analysis in the setting of genetic association studies such as candidate genes studies, genome-wide association studies (GWASs) or their replication and validation studies. Minor allele frequency (MAF), and genotypic distribution that maintains Hardy-Weinberg Equilibrium (HWE) are important characteristics of such studies. Also, the effects of the most genetic variants on complex traits are very small to moderate. Another important consideration is potentially high degree of heterogeneity in genetic effects [12]. Besides clinical and methodological differences (e.g., variation on outcome definition) across studies, genetic studies have additional sources of heterogeneity, which can be genuine (e.g., gene-local environment interaction) or artifact of the population (e.g., variation in MAF across populations) [12]. There are a few prior simulation studies (e.g., [3,6,7,10,13]) comparing the performance of multivariable (MV) and univariate (UV) methods for bivariate problems in the setting of clinical or diagnostic studies using aggregate data generation. But, none of them considered the settings typical of meta-analysis of genetic association studies.

In this study, we compared the performance of univariate (separate) vs. multivariate (joint) meta-analysis under random-effects (RE) assumption. When heterogeneity exists (which is quite likely for genetic association studies [12] as discussed above), random-effect analysis is the sensible and natural framework that can utilize the non-zero between-study correlation [3]. Although fixed-effect (FE) analysis has higher power to detect or discover disease-associated genetic variants [14,15,16], random-effects assumption is desirable for the generalization of the finding across populations. Multivariate approach theoretically offers some promise when there is moderate to high heterogeneity in true effects on correlated traits, and we wanted to assess if there is any practical advantage in different scenarios in the setting of genetic studies. We considered the following scenarios varying: 1) multivariate dimension, *p* (2-variate and 3-variate end points), 2) degrees of between-study correlation, 3) degrees of within-study correlation, 4) levels of heterogeneity, 5) average size of individual study, 6) size of meta-analysis. Each of these scenarios were analyzed under four different aggregate (summary) data availability scenarios: a) all aggregate data are available, b) all aggregate data except estimates of within-study correlations are available, hence are ignored in the meta-analysis, c) aggregate data for some studies are missing at random for end point 2, and d) aggregate data for some studies are missing informatively for end point 2. We evaluated the performance with respect to mean bias, relative mean bias percentage and root mean square error of the pooled estimate of effect and coverage probability of the 95% confidence interval of the effect for each end point via extensive simulation.

## Methods

In a meta-analysis of genetic association studies, suppose we are interested in the estimation of overall (average) effects of some factor *X* on multiple correlated quantitative traits or multiple correlated estimates at different levels of the same factor on a trait. Correlated traits could be HDL and LDL, *X* could be the number of copies of minor (mutant) allele in a genotype of a single nucleotide polymorphism (SNP) at a specific DNA locus in an individual, and the effects could be we the average increase/decrease in the traits values per one copy increase in *X* (under additive model of inheritance). Such a meta-analysis could be performed using univariate or multivariate approach.

### Meta-analysis approaches

#### Univariate (UV) meta-analysis

In the *i*th study (*i* = 1,2,…,*m*), suppose $y_{ijk}^{*}$ is the value of the *j*th phenotype (*j* = 1,2,…,*p*) from the *k*th subject (*k* = 1,2,…,*N*_*i*_), and *x*_*ik*_ is the corresponding value of *x*. Then their relationship in the original study or in a meta-analysis when individual participant data (IPD) are available can be modeled as (can include other covariates as well)

$$y_{ijk}^{*}=\;\alpha_{ij}+\beta_{ij}x_{ik}+\varepsilon_{ijk},\;\varepsilon_{ijk}\;\sim \;N\left(0,\;\sigma_{ij}^{2}\right),$$ (1)

where, *β*_*ij*_ is the true effect of *x* on the *j*th end point (phenotype) and $\sigma_{ij}^{2}$ is the error variance in study *i*. In a univariate random-effects (RE) meta-analysis, we are interested in estimating *β*_*j*_, the average of *β*_*ij*_'s of *x* on *j*th phenotype, from *m* studies. For the observed effect (estimate), $Y_{ij}{\;(=\;\hat{\beta }}_{ij})$ and its variance $s_{ij}^{2}$ for the end point *j* (obtained by, say, fitting Eq 1) in study *i*, we usually assume $Y_{ij}|\beta_{ij}\;\sim \;N(\beta_{ij},s_{ij}^{2})$ and $\beta_{ij}\;\sim \;N(\beta_{j},\;\tau_{j}^{2})$, where $\tau_{j}^{2}$ is between-study variance for the end point *j*. Hence, we can use the marginal distribution, $Y_{ij}\;\sim \;N(\beta_{j},\;s_{ij}^{2}+\tau_{j}^{2})$ for the estimation of parameters. In practice, $\tau_{j}^{2}$ is unknown and is most commonly estimated by the method of moment (MM). It can also be estimated by some likelihood based method such as restricted maximum likelihood (REML) which performs better than MM especially when number of studies is limited. The estimate of *β*_*j*_ and its variance for the end point *j* are obtained as

$${\hat{\beta }}_{j}=\;\sum_{i=1}^{m}w_{ij}\;Y_{ij}/\sum_{i=1}^{m}w_{ij}\text{ and var }\left({\hat{\beta }}_{j}\right)=1/\sum_{i=1}^{m}w_{ij},$$

where, $w_{ij}\;=\;1/\;(s_{ij}^{2}+{\hat{\tau }}_{j}^{2})$, is the weight of the *i*th study for the *j*th phenotype (*j* = 1,…,*p*). If fixed-effect (FE) of *x* is assumed (i.e., *β*_*ij*_ = *β*_*j*_ for all *i*, hence $\tau_{j}^{2}\;=\;0)$, then *β*_*j*_ is interpreted as the true effect, rather than the average effect, of *x* on the *j*th phenotype that we wish to estimate in a univariate meta-analysis. In FE analysis, ${\hat{\beta }}_{j}$ and its variance are similarly computed as above except that $w_{ij}\;=\;1/\;s_{ij}^{2}$ is used.

#### Multivariate (MV) meta-analysis

Multivariate meta-analysis is the generalization of univariate meta-analysis when *p* ≥ 2 and is theoretically a promising alternative when the *p* traits are correlated. In individual studies, we can jointly model the multiple phenotypes as

$$y_{ik}^{*}=\alpha_{i}\;+\beta_{i}x_{ik}\;+\varepsilon_{ik},\;\varepsilon_{ik}\;\sim \;N_{p}\left(0,\;\Psi_{i}\right),$$ (2)

where ***β***_*i*_ = (*β*_*i*1_,*β*_*i*2_,…,*β*_*ip*_)^*t*^ is the *p*-dimensional true effects of *x* jointly on all *p* phenotypes and ***Ψ***_**i**_ is the *p* × *p* residual covariance matrix in study *i*. In random-effect multivariate meta-analysis, we are interested in simultaneously estimating the average joint effects vector ***β*** = (*β*_1_,*β*_2_,…,*β*_*p*_)^*t*^ of *x* on *p* phenotypes in overall population from *m* studies. Let $Y_{i}\;=\;{\left(Y_{i1},\;Y_{i2},\;\ldots ,\;Y_{ip}\right)}^{t}\;=\;{\left({\hat{\beta }}_{i1},\;{\hat{\beta }}_{i2},\ldots ,{\hat{\beta }}_{ip}\right)}^{t}$ be the joint observed (estimated) effects and ***S***_*i*_ be covariance matrix of ***Y***_*i*_ in study *i*. Under RE model, let’s assume that ***β***_*i*_ **∼** *MVN*_*p*_(***β*,Σ**) and its estimates ***Y***_*i*_ ***|β***_*i*_ **∼** *MVN*_*p*_ **(*β***_*i*_, **S**_*i*_ **)** in study *i*. Then, we can use the marginal distribution of the joint estimate in study *i* is ***Y***_*i*_ **∼** *MVN*_*p*_ **(*β*, Σ + S**_*i*_ **)** to estimate the parameter **Σ** and then compute the estimate of ***β***. Here, **Σ**, the between-study covariance matrix represents population variation in the studies’ true underlying effects, while ***S***_*i*_, the within-study covariance matrix represents variation in the *i*th study’s results due to repeated sampling or chance. For instance, in bivariate problem:

$$\text{Linear model for IPD in study }i:\;\left(\begin{array}{c} y_{i1k}^{*}\\ y_{i2k}^{*} \end{array}\right)=\left(\begin{array}{c} \alpha_{i1}\\ \alpha_{i2} \end{array}\right)+\left(\begin{array}{c} \beta_{i1}\\ \beta_{i2} \end{array}\right)x_{ik}+\left(\begin{array}{c} \varepsilon_{i1k}\\ \varepsilon_{i2k} \end{array}\right)$$ (3)

$$\text{Summary data in study }i:\;Y_{i}=\left(\begin{array}{c} Y_{i1}\\ Y_{i2} \end{array}\right)=\left(\begin{array}{c} {\hat{\beta }}_{i1}\\ {\hat{\beta }}_{i2} \end{array}\right),\;S_{i}=\left(\begin{array}{cc} s_{i1}^{2} & \rho_{wi}s_{i1}s_{i2}\\ \rho_{w}s_{i1}s_{i2} & s_{i2}^{2} \end{array}\right)$$ (4)

$$\text{Parameters to be estimated}:\;\beta =\left(\begin{array}{c} \beta_{1}\\ \beta_{2} \end{array}\right),\;\Sigma =\left(\begin{array}{cc} \tau_{1}^{2} & \rho_{b}\tau_{1}\tau_{2}\\ \rho_{b}\tau_{1}\tau_{2} & \tau_{2}^{2} \end{array}\right)$$ (5)

$$\text{Marginal model}:\;\left(\begin{array}{c} Y_{i1}\\ Y_{i2} \end{array}\right)\sim MVN_{2}\;\left(\left(\begin{array}{c} \beta_{1}\\ \beta_{2} \end{array}\right),\;\left(\begin{array}{cc} \tau_{1}^{2}+s_{i1}^{2} & \rho_{b}\tau_{1}\tau_{2}+\rho_{wi}s_{i1}s_{i2}\\ \rho_{b}\tau_{1}\tau_{2}+\rho_{wi}s_{i1}s_{i2} & \tau_{2}^{2}+s_{i2}^{2} \end{array}\right)\right)$$ (6)

Here, $\tau_{1}^{2}$ and $\tau_{2}^{2}$, known as between-study variances, are the variances of *β*_*i*1_ and *β*_*i*2_ across studies, and $s_{i1}^{2}$ and $s_{i2}^{2}$, known as the within-study variances, are the variances of *Y*_*i*1_ and *Y*_*i*2_ within study *i*, respectively. The between-study correlation, *ρ*_*b*_, is the correlation between *β*_*i*1_ and *β*_*i*2_ across studies (or populations) and the within-study correlation, *ρ*_*wi*_, is the correlation between *Y*_*i*1_ and *Y*_*i*2_ within study *i*.

In a meta-analysis, the within-study covariance matrix ***S***_*i*_ (i.e., $\rho_{wi},\;s_{i1}^{2}and\;s_{i2}^{2}$ for *p* = 2) is assumed to be known in all studies (*i* = 1,2,…,*m*). However, in real meta-analysis it is typically estimated from individual participant data, if accessible, by fitting the Eq 2 in each study. If IPD is not available in all studies, then the estimates of *ρ*_*wi*_’s might not be available in the corresponding published studies for the reasons: some of the published studies might report the aggregate data on ${(Y}_{ij},\;s_{ij}^{2}),\;j\;=\;1,\ldots ,p,$ that were obtained by fitting Eq 1 separately for each trait, and some might report only ${(Y}_{ij},\;s_{ij}^{2}),\;$ but not ${\hat{\rho }}_{wi}$'s even if those aggregate data were obtained by fitting Eq 2 jointly on all traits. In such case, *ρ*_*wi*_’s might have to be inferred or estimated indirectly for multivariate meta-analysis [6] or different multivariate meta-analytic technique that does not require ${\hat{\rho }}_{wi}$'s can be employed [8]. For a RE meta-analysis, **Σ** (e.g., three parameters $\tau_{1}^{2},\;\tau_{2}^{2}$, and *ρ*_*b*_ if *p* = 2) is estimated before computing the estimate of ***β*** (i.e., two more parameters *β*_1_ and *β*_2_ for *p* = 2). But, in univariate analysis only one parameter $\tau_{j}^{2}\;(j\;=\;1,2,\ldots ,p)$ is first estimated separately for the *j*th endpoint before computing the estimate of *β*_*j*_. Restricted maximum likelihood (REML) method is commonly used for estimation of **Σ**, assuming multivariate normality of random effects (i.e., ***β***_*i*_ **∼** *MVN*_*p*_(***β*,Σ**)). REML generally produces smaller variance estimates within the realistic parameter space compared to the method of maximum likelihood [3,4]. However, when multivariate normality is not met or is questionable, multivariate method of moment (MMM) [17] or a method based on the theory of U statistics may provide more unbiased estimate of **Σ** [18]. Then the estimate of ***β*** and its variance are obtained as

$$\hat{\beta }\;=\;{\left(\sum_{i=1}^{m}W_{i}\right)}^{-1}\;\left(\sum_{i=1}^{m}W_{i}Y_{i}\right),\text{ and var }(\hat{\beta })\;=\;{\left(\sum_{i=1}^{m}W_{i}\right)}^{-1}\text{ where }W_{i}\;=\;{\left(S_{i}+\;\hat{\Sigma }\right)}^{-1}$$

Under multivariate fixed-effect (FE) meta-analysis model, the between study heterogeneity is assumed to be absent, i.e., **Σ = 0** is assumed.

### Simulation and estimation methods

#### Meta-analysis of estimated aggregate data from IPD data generation

We first generated the IPD data for a range of scenarios varying the study level parameters such as average sample sizes, number of studies, etc. and estimated the summary data (i.e., effects estimates and their variances and correlation(s) within a study) in each study to ensure that we pool realistic summary data typical of genetic association studies. We then pooled them over all studies, thus performing a two-stage IPD meta-analysis. Estimating aggregate data by generating IPD in individual studies (rather than directly sampling aggregate data using some distributions) has another advantage that it also allows us to vary study level parameters such as sizes of individual studies, and MAF of a genetic variant across studies and maintain the Hardy-Weinberg Equilibrium (HWE) within each study, etc. This in turn allows us to assess the impact of such study level parameters on the performance of methods. However, we also compared the performance of two approaches by directly generating (sampling) aggregate data from some reasonable distributions.

In the first stage of IPD meta-analysis, we considered a meta-analytic problem of estimating the effect of *X* (*x* = 0,1,2), with two traits (*p* = 2) and three traits (*p* = 3). We considered the minor allele frequency (MAF), *f* = 0.20 at the locus. We considered different scenarios with a set of number of studies (*m*), and total meta-analysis size (*N*), as *m* = 5 and *N* = 5000; *m* = 5 and *N* = 10000; *m* = 10 and *N* = 10000; *m* = 15 and *N* = 20000; *m* = 30 and *N* = 30000, with the average study size, *n* = *N*/*m*. To approximate the practical situation where all studies will not be of equal size and the distribution of minor allele will not be the same across all populations, we considered variable study size (*N*_*i*_) around *n* and slightly variable MAF (*f*_*i*_) around *f* across studies. The distribution of *X* maintained HWE at *p*-value **≥** 0.001 in HWE test in each study.

The study-wise effect vector ***β***_***i***_ = (*β*_*i*1_,*β*_*i*2_,…,*β*_*ip*_)^*t*^ were simulated from *N*_*p*_(***β***,**Σ**), where ***β*** = (*β*_1_,*β*_2_ **)** ^t^ for *p* = 2 and ***β*** = (*β*_1_,*β*_2_,*β*_*3*_)^*t*^ for *p* = 3. We considered small to modest genetic effect size β_*j*_ for trait *j* from a pool = {0.10,0.15,0.20,0.25,0.30,0.40}. For instance, *β*_1_ = 0.10 and *β*_2_ = 0.10 in a scenario with *p* = 2, and *β*_1_ = 0.20, *β*_2_ = 0.30 and *β*_3_ = 0.30 in another scenario with *p* = 3 was considered. Since the vast majority of causal SNPs might contribute only little and only a few of them contribute considerably to heritability of a quantitative complex trait, this pool of β_*j*_ represents a reasonable spectrum of heritability, $h_{j}^{2}\;\approx$ {0.003 to 0.050} due to an individual causal SNP with *f* = 0.20 and small to modest effect (where $\beta_{j}\;=\;\sqrt{h_{j}^{2}/2f(1-f)}$, under additive genetic risk model [19]). For such effect sizes and average study size, it is critical to choose realistic values of **Σ** for simulation. We first calculated an approximate value of average within-study variance, $s_{j}^{2}\;=\;s^{2}$ (for all *j* = 1,2…,*p*) of the estimate of β_*j*_ in a study with the average size *n* = *N*/*m* and MAF distribution strictly under HWE for *f* = 0.20 (see S1 File for details). Then we obtained $\tau_{j}^{2}$ as $\tau_{j}^{2}\;=\;s_{j}^{2}/3,\;s_{j}^{2},\;3s_{j}^{2}$ (for all *j*) for the between-study heterogeneity, *I* ^2^ = 25%,50%, and 75%, respectively. Here, $I^{2}\;=\;\tau_{j}^{2}/(\tau_{j}^{2}+s_{j}^{2})\;$ is the proportion of total variance due to true (between-study) heterogeneity [20]. The covariance elements of **Σ** are obtained as $\tau_{jj'}\;=\;\rho_{bjj'}\tau_{j}\tau_{j'},\;(j\neq j^{'}\;=\;1,2,\ldots ,p)$, where we chose a *ρ*_*bjj*_, from a pool {0.20,0.50,0.60,0.70,0.75}.

In the first stage of IPD meta-analysis, we simulated IPD trait values $y_{ik}^{*}\;=\;{\left(y_{i1k}^{*},\;y_{i2k}^{*},\;\ldots ,\;y_{ipk}^{*}\right)}^{t}$, for the *k*th subject (*k* = 1,2,…,*N*_*i*_) with genotype *x* = *x*_*ik*_ (*x* = 0,1,or 2) from study *i* (*i* = 1,2,…,*m*) for *p* = 2 or *p* = 3 scenario as in [21,22,23]

$$y_{ik}^{*}\sim N_{p}\;(\alpha_{i}+\beta_{i}x_{ik},\;\Psi_{i}),$$

where, ***α***_*i*_ is a *p* × 1 vector of intercepts (baseline effects on *j* traits when *x* = 0), ***β***_*i*_ is the *p* × 1 vector of the true effect of *x* and **Ψ**_***i***_ is a *p* × *p* residual variance matrix with *p* diagonal elements as the error variances ($\sigma_{\varepsilon_{ij}}^{2}$) of individual observations on each of *p* traits and off-diagonal elements as corresponding covariances ($\sigma_{\varepsilon_{ijj'}}\;=\;\rho_{w_{ijj'}}\sigma_{\varepsilon_{ij}}\sigma_{\varepsilon_{ij'}}$), in study *i*. We chose a within-study correlation, $\rho_{w_{jj'}}$ from the pool {0,0.3,0.5,0.75}. We fixed ***α***_*i*_ = (1,5)^*t*^ for *p* = 2 and ***α***_*i*_ = (1,5,10)^*t*^ for *p* = 3 for all *x* and *m*, and let $\sigma_{\varepsilon_{ij}}^{2}≅1$ for all *x* genotypes, *p* traits and *m* studies to ensure the identifiability of the model for comparison purpose in our simulation [21,22,23]. (See 'Adequacy of chosen simulation parameters and accuracy of estimation' section in S1 File for how **Ψ**_***i***_ was constructed.).

The study-wise estimates $Y_{i}\;=\;{\hat{\beta }}_{i}$ and their variances matrix ***S***_*i*_ (within-study variances $s_{ij}^{2}$ and covariances ${s_{ijj'}\;=\;\hat{\rho }}_{w_{ijj'}}s_{ij}s_{ij'},\;j\neq j^{'}\;=\;1,2,\ldots ,p$) in study *i* (*i* = 1,2,…,*m*) were simultaneously obtained by fitting multivariate linear regression of $y_{ik}^{*}$ on *x* in each study.

Simulation methods and scenarios for two-stage IPD meta-analysis are summarized in Table 1. Adequacy of the choice of simulation parameters mimicking the setting of genetic studies was assessed based on GWAS catalogue [24] and provided in Section A in S1 File. The steps in the first stage of IPD meta-analysis are summarized in Section B in S1 File.

**Table 1:** Simulation scenarios and methods for IPD data generation.

Parameters	Assumed values
Suffix	*i* = 1,2,…,*m* studies; *j*,*j’* = 1,…,*p* endpoints; *k* = 1,…,*N*_*i*_ subjects in study *i*
No. of replications	*R* = 5000
No. of end points	*p* = 2, 3
No. of studies (*m*) and total subjects (*N*)	*m* = 5 and *N* = 5000; *m* = 5 and *N* = 10000; *m* = 10 and *N* = 10000; *m* = 15 and *N* = 10000; *m* = 30 and *N* = 30000
MAF	*f* = 0.20
Size of study *i*	*N*_*i*_ ∼ *uniform*(*N*/*m* − *N*/2*m*, *N*/*m* + *N*/2*m*), $N_{i}’$ s were proportionally adjusted so that $\sum_{i\;=\;1}^{m}N_{i}\;=\;N$.
MAF in study *i*	*f*_*i*_∼*N*(*mean* = *f*, *sd* = *f*/5), fi' s were adjusted so that $\sum_{i\;=\;1}^{m}f_{i}/m\;=\;f$.
No. of minor allele	*x* = 0,1,2
Genotype distribution	In study *i*, for a SNP with MAF *f*_*i*_ the genotype of *N*_*i*_ subjects were sampled for *N*_*i*_ times with replacement from *x* = {0,1,2} with corresponding probabilities as the frequencies {${\left(1-f_{i}\right)}^{2},\;2f_{i}\left(1-f_{i}\right),\;f_{i}^{2}$} (of genotypes distribution strictly under HWE). HWE in each study was assessed through HWE exact test, and sample was redrawn if HWE was not met (i.e., if P-value < 0.001) in the study.
Heritability	$h_{j}^{2}\;=\;\{0.003\;to\;0.05\}$ for a causal SNP
SNP effects	$\beta_{j}\;=\;\sqrt{h_{j}^{2}/2f(1-f)}\;\approx$ {0.1 to 0.4} for *f* = 0.20 and range of $h_{j}^{2}$
SNP joint effect	***β*** = (*β*_1_,…,*β*_*p*_)^*t*^; e.g., ***β*** = (0.1,0.1)^*t*^ for *p* = 2, and ***β*** = (0.20,0.30,0.30)^*t*^ for *p* = 3
Between-study correlation	*ρ*_*bjj*_, = {0.2,0.5,0.6,0.7,0.75}; e.g., *ρ*_*b*_ = .5 for *p* = 2, and *ρ*_*b*12_ = .7, *ρ*_*b*13_ = .5, *ρ*_*b*23_ = .6 for *p* = 3
Heterogeneity	I^2^ = 25%,50%,75% (low, moderate, and high heterogeneity)
Between study variance, $\tau_{j}^{2}$	First, an average of within-study variance $s_{j}^{2}$ of the estimate of *β*_*j*_ was obtained by generating IPD data in an average size study *N*/*m* with the distribution of *x* strictly under HWE for a MAF *f* = 0.20 and then fitting the linear regression model $y_{ijk}^{*}\;=\;\alpha +\beta_{j}x+\varepsilon_{ik}\;(\varepsilon_{ik}\sim N\left(0,\;\sigma_{\varepsilon }^{2=}\text{1}\right)\alpha =\text{1})$ 1000 times. (It would roughly be $s_{j}^{2}\approx {\left(X^{t}X\right)}^{-1}\sigma_{\varepsilon }^{2}\approx m\sigma_{\varepsilon }^{2}/Nvar(X)$ in a data). Then, a rough value of $\tau_{j}^{2}\;=\;s_{j}^{2}I^{2}/(1-I^{2})$ (for all *j*) was obtained for each level of *I* ^2^ and finally **Σ** to be used in the scenario was constructed from *ρ*_*bjj*_,’s and $\tau_{j}^{2}$’s.
Study-wise effects	**β**_*i*_ were sampled from N_*p*_(**β**,**Σ**)
Within-Study correlation	$\rho_{w_{jj'}}\;=\;\{0,0.3,0.5,\;0.7\}$. In study *i*, we considered *ρ*_*wijj*_, = *ρ*_*wjj*_,. E.g., *ρ*_*wi*_ = .3 for *p* = 2, and *ρ*_*wi*12_ = .3, *ρ*_*wi*13_ = .3,*ρ*_*wi*23_ = .5, for *p* = 3 for all *i*.
Baseline effect (when *x* = 0)	***α***_***i***_ = (1,5)^*t*^ for *p* = 2 and ***α***_*i*_ = (1,5,10)^*t*^ for *p* = 3 for all *i*.
Residual variance matrix	Diagonal element of **Ψ**_*i*_ are close to 1 ($\sigma_{\varepsilon_{ij}}^{2}≅1$) and off-diagonal element $\sigma_{\varepsilon_{ijj'}}\;=\;\rho_{w_{jj'}}\sigma_{\varepsilon_{ij}}\sigma_{\varepsilon_{ij'}}.$
IPD data generation	$y_{ik}^{*}$ were sampled from N_*p*_ **(*α***_i_ + **β**_*i*_ *x*,**Ψ**_*i*_ **)**
Summary data in study *i*	$y_{i}\;=\;{\hat{\beta }}_{i}$ and ***S***_*i*_ are estimated by fitting multivariate linear regression of $y_{ik}^{*}$ on *x* in study *i*.

Abbreviations: IPD, individual participant data; MAF, minor allele frequency; HWE, Hardy-Weinberg Equilibrium; SNP, single nucleotide polymorphism.

In the second stage of IPD meta-analysis, we meta-analyzed the estimated summary data ***Y***_*i*_’s and ***S***_*i*_’s across all studies in each scenario performing both multivariate and univariate meta-analyses under random-effects assumption. For each combination of parameters, we considered four scenarios related to the availability of aggregate data: 1) complete data scenario, 2) complete data scenario but ${\hat{\rho }}_{w_{ijj^{'}}}$ were ignored, 3) missing at random scenario, 4) missing informatively scenario. Under *complete data scenario*, we utilized all summary data including $\rho_{w_{jj^{'}}}$ when applicable for all end points. Under *complete data scenario with ignoring ${\hat{\rho }}_{w_{ijj^{'}}}$*, we set ${\hat{\rho }}_{w_{ijj^{'}}}\;=\;0$ (assuming ${\hat{\rho }}_{w_{ijj^{'}}}$ to be 0’s for all possible *j* **≠** *j*′ pairs) and re-meta-analyzed under MV framework. This allows us to assess the impact of ignoring within-study correlation when they are missing (not reported) in some or all studies and investigators choose to ignore such correlation rather than inferring them indirectly [6] or using alternative techniques [8] in multivariate meta-analysis. Under *missing at random (MAR) scenario*, we assumed that about 30% (*m*′ = 0.3*m*, rounded to the nearest integer) of studies had randomly missing summary data for end point 2 and chose those studies randomly. Random missing is likely in a meta-analysis of genetic studies if investigators in some studies do not consider estimating and reporting the risk of a genetic variant on some trait(s) that are of interest in the meta-analysis. Under *sing informatively* (*MIF*) *scenario*, we assumed that about 30% (*m*′ = 0.3*m*) of the studies had informatively missing (for some reason) summary data for end point 2. This is a typical scenario representing ‘publication bias’ in a meta-analysis of genetic studies, where investigators might not report or journal might not publish insignificant genetic association of a variants with some trait in some studies, whereas significant genetic association in any direction (irrespective whether it is protective or risk) is still more likely to be reported and published. Under this scenario, we identified the first *m*′ smallest $\chi_{1}^{2}\;=\;{{(y}_{i2}/s_{i2})}^{2}$ out of *m* studies for end point 2 and considered them to be missing.

In each data availability scenario, summary data for all end points were jointly meta-analyzed by MV approach, where the missing summary data for end point 2 were imputed in both MAR and MIF scenario before the meta-analysis. For each of the *m*′ missing studies (*l* = 1,2,…,*m*′), we considered *y*_*l*2_ = 0, *y*_*l*2*j*′_ = 0 for *j*′ **≠** 2 (i.e., setting ${\hat{\rho }}_{w_{l2j^{'}}}\;=\;0$) and $s_{l2}^{2}\;=\;10$. This is a conservative imputation strategy that gives a too small weight to the missing (and imputed) within-study estimate of 0 compared to non-missing end point(s) in a study in order to utilize all non-missing end point data in MV approach. In both missing data scenarios, we utilized ${\hat{\rho }}_{w_{ijj'}}$’s available from all non-missing studies. Under UV approach, summary data for each end point were meta-analyzed separately, where all data for non-missing end point and available data for missing end point 2 were used. Estimation steps for different data availability scenarios are summarized in Section B in S1 File.

We fitted the multivariate RE meta-analysis model using *mvmeta* package and separate univariate RE meta-analysis model for each end point) using *metafor* package in R language. The estimates of **Σ** in MV meta-analysis and $\tau_{j}^{2}$ in UV meta-analysis were obtained by REML approach. We used $t_{m_{j}-1,\;.025}$ value to construct the 95% confidence interval of each effect parameter, β_*j*_ (*j* = 1,…,*p*) [3,25]. We repeated each scenario for *R* = 5000 times (number of replications). (See Section B in S1 File for summary of estimation steps for the meta-analysis of IPD data.)

We compared the performance of MV and UV approaches with respect to of mean bias, relative mean biases percentage (% bias), and RMSE in of each of ${\hat{\beta }}_{j},\;{\hat{\tau }}_{j}^{2}$ and ${\hat{\rho }}_{bjj'}$, and coverage probability of 95% confidence intervals of β_*j*_ (*j* = 1,…,*p*). We also compared the percentage of times ${\hat{\tau }}_{j}^{2}$ was estimated at parameter boundary (i.e., ${\hat{\tau }}_{j}^{2}\;=\;0$) by both UV and MV approaches and ${\hat{\rho }}_{bjj'}$ estimated at the boundary of parameter space (i.e., ${\hat{\rho }}_{bjj'}\;=\;-1,\;+1$, |1|) by the MV method, where we defined ${\hat{\tau }}_{j}^{2}\;=\;0$ if ${\hat{\tau }}_{j}^{2}\;<\;.00005$ and ${\hat{\rho }}_{bjj'}\;=\;|1|$ if $|{\hat{\rho }}_{bjj'}|>.9995$ [17,18]. We also defined under and over estimations of ${\hat{\rho }}_{bjj'}$ as ${\hat{\rho }}_{bjj'}<-0.95$ and ${\hat{\rho }}_{bjj'}>0.95$ since ${\hat{|\rho }}_{bjj'}|>0.95$ is an indication of unstable estimation [8].

#### Meta-analysis of directly sampled aggregate data

Additionally, we compared the performance of multivariate and univariate approaches via simulation in a few specific scenarios by directly generating (sampling) aggregate data (***Y***_*i*_ and ***S***_*i*_) in each study (as opposed to estimating them in the first-stage of the IPD meta-analysis described above). For this, we considered bivariate case with ***β*** = (*β*_1_ = 0.1, *β*_2_ = 0.1)^*t*^. We used the same **Σ** (i.e., $\tau_{j}^{2}$'s and *ρ*_*b*_) and average $s_{j}^{2}$ as $\tau_{j}^{2}$ for *I* ^2^ = 50% as in two-stage IPD meta-analysis for the similar scenario. To facilitate the direct generation of realistic *S*_*i*_, we relied on the distribution of summary estimates (i.e., average SD of *S*_*ij*_'s (*j* = 1, 2) and ${\hat{\rho }}_{wi}$’s, and correlation(*S*_*i*1_, *S*_*i*2_) across studies) observed in the analysis of IPD data. (See Section C in S1 File for details). Then we directly generated ***Y***_*i*_ = (*Y*_*i*1_, *Y*_*i*2_)^*t*^ from its marginal distribution ***Y***_*i*_ *∼N*_2_(***β***, **Σ** + ***S***_*i*_) provided in Eq 6. Thus, our data generation process is slightly different and more realistic than previous simulation study (e.g., [6]) comparing the multivariate and univarite approaches in clinical setting in that we maintained the likely correlation between within-study variances between two end points (as they are likely to be similar from the same study) and also considered variable within-study correlations across studies.

The directly generated summary data were then meta-analyzed using multivariate and univariate approaches with RE assumption as in the second stage of IPD meta-analysis described above.

## Simulation Results

**Fig 1:**
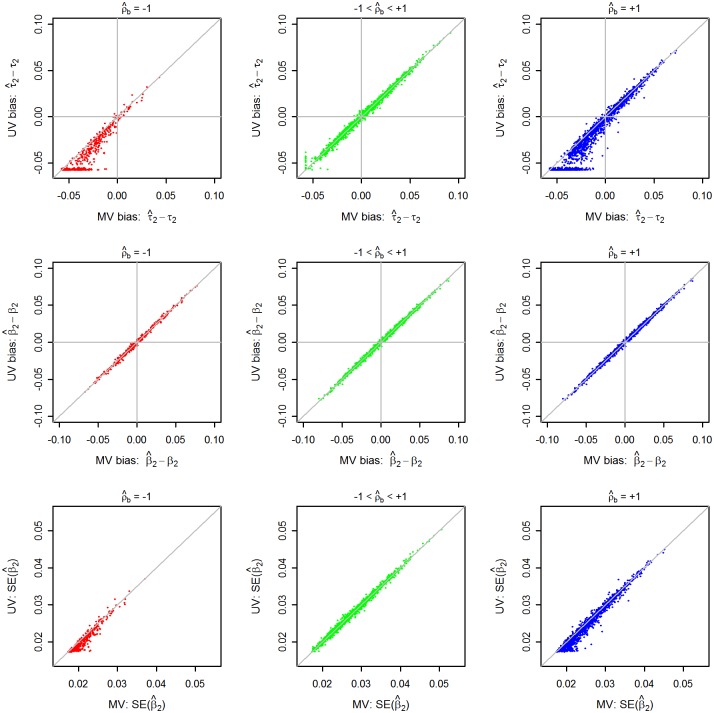
Biases in the estimates of τ_2_, and biases and SEs of the pooled estimates of *β*_2_ from multivariate vs. univariate approaches by whether or not *ρ*_*b*_ is estimated at parameter boundary in 5000 replications in complete summary data scenario. Scenario: $N\;=\;10000,\;m\;=\;10,\;MAF\;=\;0.20,\;\beta_{1}\;=\;0.3,\;\beta_{2}\;=\;0.4,\;\tau_{1}^{2}\;=\;\tau_{2}^{2}\;=\;0.0033;\;I^{2}\;=\;50\%$, *ρ*_*b*_ = 0.75, *ρ*_*w*_ = 0.5. Symbols and abbreviations: *N*, total subjects; *m*, number of studies, *β*_2_ and *τ*_2_, average effect and between-study standard deviation of true study-wise effects for end point 2, respectively; *I* ^2^ = degree of between-study heterogeneity; *ρ*_*b*_ and *ρ*_*w*_, true between-and within-study correlations, respectively; MAF, minor allele frequency; SE, standard error; MV, multivariate approach; UV, univariate approach.

Comparative performance of multivariate and univariate RE meta-analytic methods in certain key scenarios based on estimation of summary data through IPD data generation and analysis are presented on Tables 2–5 and Fig 1. More results are presented on Tables A-F in S2 File and S1–S5 Figs. Comparative results based on the directly sampled aggregate data are presented in Tables G-J in S2 File. In the supplementary tables in S2 File, the results at low heterogeneity (i.e., when I^2^ = 25%) at which multivariate approaches are thought to offer no clear benefit, are also presented.

**Table 2:** Relative mean bias percentage, RMSE and coverage probability when N = 10000, m = 10, *β*_1_ = 0.1, *β*_2_ = 0.1, *ρ*_*b*_ = 0.5, *ρ*_*w*_ = 0.3.

	Summary		Effects						Heterogeneity				Correlation			
	data		% Bias		RMSE^b^		Coverage		% Bias		RMSE^b^		% Bias	%(${\hat{\rho }}_{b})$		
Method	scenario	*I* ^2^,^a^	${\hat{\beta }}_{1}$	${\hat{\beta }}_{2}$	${\hat{\beta }}_{1}$	${\hat{\beta }}_{2}$	${\hat{\beta }}_{1}$	${\hat{\beta }}_{2}$	${\hat{\tau }}_{1}^{2}$	${\hat{\tau }}_{2}^{2}$	${\hat{\tau }}_{1}^{2}$	${\hat{\tau }}_{2}^{2}$	(${\hat{\rho }}_{b}$)	-1	+1	|1|
MV	COM	50%	0	1	0	0	95.7	96.2	6	3	-3	-3	-17	11.8	30.5	42.3
UV	COM		0	1	0	0	95.3	95.8	3	0	0	0	—	—	—	—
MV	COM^c^		0	1	0	0	95.9	96.2	8	8	-3	-3	31	6.1	49.3	55.4
MV	MAR		0	0	0	0	95.9	95.0	6	8	-3	0	-22	15.7	38.6	54.3
UV	MAR		0	0	0	0	95.3	96.3	3	3	0	0	—	—	—	—
MV	MIF		0	27	0	-5	95.7	80.7	6	-19	-3	-3	-34	21.3	41.9	63.3
UV	MIF		0	31	0	0	95.3	81.5	3	-25	0	0	—	—	—	—
MV	COM	75%	0	0	0	0	95.0	94.9	0	-1	0	0	-9	2.1	7.7	9.8
UV	COM		0	0	0	0	95.0	94.8	0	-1	0	0	—	—	—	—
MV	COM^c^		0	0	0	0	95.0	94.8	1	0	0	0	14	1.3	15.6	16.9
MV	MAR		0	0	0	0	95.0	93.2	1	1	0	1	-12	4.8	15.9	20.7
UV	MAR		0	0	0	0	95.0	95.4	0	0	0	0	—	—	—	—
MV	MIF		0	29	0	-4	95.1	79.1	1	6	0	0	-14	7	18.1	25.1
UV	MIF		0	35	0	0	95.0	79.9	0	5	0	0	—	—	—	—

Abbreviation: RMSE, root mean square error; MV, multivariate meta-analysis; UV, Univariate meta-analysis; COM, complete data scenario; MAR, end point 2 missing at random for 30% studies; MAR, end point 2 missing informatively for 30% studies.

^a^The between-study variances for both end-points $\tau_{j}^{2},\;j\;=\;1,2$ are: $\tau_{j}^{2}$ = 0.0036 for *I* ^2^ = 50%, $\tau_{j}^{2}$ = 0.0108 for *I* ^2^ = 75%.

^b^RMSE of estimates by MV method are expressed as % smaller (-) or larger (+) of corresponding estimates by UV method.

^c${\hat{\rho }}_{w}\;$^ ignored.

**Table 3:** Relative mean bias percentage, RMSE and coverage probability when N = 20000, m = 15, *β*_1_ = 0.1, *β*_2_ = 0.1, *ρ*_*b*_ = 0.6, *ρ*_*w*_ = 0.3.

	Summary		Effects						Heterogeneity				Correlation			
	data		% Bias		RMSE^b^		Coverage		% Bias		RMSE^b^		% Bias	%(${\hat{\rho }}_{b})$		
Method	scenario	*I* ^2^,^a^	${\hat{\beta }}_{1}$	${\hat{\beta }}_{2}$	${\hat{\beta }}_{1}$	${\hat{\beta }}_{2}$	${\hat{\beta }}_{1}$	${\hat{\beta }}_{2}$	${\hat{\tau }}_{1}^{2}$	${\hat{\tau }}_{2}^{2}$	${\hat{\tau }}_{1}^{2}$	${\hat{\tau }}_{2}^{2}$	(${\hat{\rho }}_{b}$)	-1	+1	|1|
MV	COM	50%	0	0	0	0	94.8	95.2	0	4	0	-5	-5	5	27	32
UV	COM		0	0	0	0	94.7	95.1	0	4	0	0	—	—	—	—
MV	COM^c^		0	0	0	0	95.2	95.4	7	7	0	-5	34	1.6	52.4	53.9
MV	MAR		0	0	0	-3	95.0	94.7	4	7	0	0	-12	8.4	35.7	44.1
UV	MAR		0	0	0	0	94.7	95.6	0	4	0	0	—	—	—	—
MV	MIF		0	27	0	-9	94.9	66.2	4	-41	0	-4	-26	16.2	45.2	61.4
UV	MIF		0	32	0	0	94.7	63.1	0	-48	0	0	—	—	—	—
MV	COM	75%	0	0	0	0	94.7	94.5	0	1	0	0	-3	0.3	3.2	3.5
UV	COM		0	0	0	0	94.8	94.5	0	1	0	0	—	—	—	—
MV	COM^c^		0	0	0	0	94.8	94.5	1	2	0	0	15	0.1	10	10.1
MV	MAR		0	0	0	-3	94.7	93.4	1	2	0	0	-4	1.1	10	11.1
UV	MAR		0	0	0	0	94.8	95.0	0	2	0	0	—	—	—	—
MV	MIF		0	30	0	-11	94.7	71.4	1	-5	0	-3	-11	3.7	13.2	16.9
UV	MIF		0	39	0	0	94.8	69.4	0	-5	0	0	—	—	—	—

Abbreviation: RMSE, root mean square error; MV, multivariate meta-analysis; UV, Univariate meta-analysis; COM, complete data scenario; MAR, end point 2 missing at random for 30% studies; MAR, end point 2 missing informatively for 30% studies.

^a^The between-study variances for both end-points $\tau_{j}^{2},\;j\;=\;1,2$ are: $\tau_{j}^{2}$ = 0.0027 for *I* ^2^ = 50%, $\tau_{j}^{2}$ = 0.0082 for *I* ^2^ = 75%.

^b^RMSE of estimates by MV method are expressed as % smaller (-) or larger (+) of corresponding estimates by UV method.

^c${\hat{\rho }}_{w}\;$^ ignored.

**Table 4:** Relative mean bias percentage, RMSE and coverage probability when N = 30000, m = 30, *β*_1_ = 0.1, *β*_2_ = 0.2, *ρ*_b_ = 0.6, *ρ*_w_ = 0.3.

	Summary		Effects						Heterogeneity				Correlation			
	data		% Bias		RMSE^b^		Coverage		% Bias		RMSE^b^		% Bias	%(${\hat{\rho }}_{b})$		
Method	scenario	*I* ^2^,^a^	${\hat{\beta }}_{1}$	${\hat{\beta }}_{2}$	${\hat{\beta }}_{1}$	${\hat{\beta }}_{2}$	${\hat{\beta }}_{1}$	${\hat{\beta }}_{2}$	${\hat{\tau }}_{1}^{2}$	${\hat{\tau }}_{2}^{2}$	${\hat{\tau }}_{1}^{2}$	${\hat{\tau }}_{2}^{2}$	(${\hat{\rho }}_{b}$)	-1	+1	|1|
MV	COM	50%	0	0	0	0	95.1	95.2	0	3	0	0	1	0.7	9.4	10.1
UV	COM		0	0	0	0	95.1	95.3	0	3	0	0	—	—	—	—
MV	COM^c^		0	0	0	0	95.3	95.4	6	6	0	0	40	0.1	39.5	39.6
MV	MAR		0	0	0	-2	95.1	94.8	0	3	0	0	-2	1.9	16.3	18.2
UV	MAR		0	0	0	0	95.1	95.3	0	3	0	0	—	—	—	—
MV	MIF		0	27	0	-12	95.2	57.8	0	-31	0	-4	-9	6.4	26.8	33.2
UV	MIF		0	32	0	0	95.1	49.7	0	-33	0	0	—	—	—	—
MV	COM	75%	0	0	0	0	94.8	94.5	1	0	0	0	-2	0	0.1	0.1
UV	COM		0	0	0	0	94.9	94.4	1	0	0	0	—	—	—	—
MV	COM^c^		0	0	0	0	94.9	94.4	1	1	0	0	15	0	1.7	1.7
MV	MAR		0	0	0	-3	94.8	94.1	1	1	0	2	-2	0	0.7	0.7
UV	MAR		0	0	0	0	94.9	94.5	1	1	0	0	—	—	—	—
MV	MIF		0	26	0	-16	94.8	76.1	1	6	0	-4	-1	0.1	1	1.1
UV	MIF		0	36	0	0	94.9	69.9	1	6	0	0	—	—	—	—

Abbreviation: RMSE, root mean square error; CI, confidence interval; MV, multivariate meta-analysis; UV, Univariate meta-analysis; COM, complete data scenario; MAR, end point 2 missing at random for 30% studies; MAR, end point 2 missing informatively for 30% studies.

^a^The between-study variances for both end-points $\tau_{j}^{2},\;j\;=\;1,2$ are: $\tau_{j}^{2}$ = 0.0036 for *I* ^2^ = 50%, $\tau_{j}^{2}$ = 0.0109 for *I* ^2^ = 75%.

^b^RMSE of estimates by MV method are expressed as % smaller (-) or larger (+) of corresponding estimates by UV method.

^c${\hat{\rho }}_{w}\;$^ ignored.

**Table 5:** Relative mean bias percentage, RMSE, and coverage probability when N = 10000, m = 10, *β*_1_ = 0.2, *β*_2_ = 0.3, *β*_3_ = 0.3, *β*_*b*12_ = 0.6, *β*_*b*13_ = 0.7, *ρ*_*b*23_ = 0.6, *ρ*_*w*12_ = *ρ*_*w*13_ = *ρ*_*w*23_ = 0.

	Summary		Effects									Heterogeneity						Correlation					
	data		% Bias			RMSE^b^			Coverage			% Bias			RMSE^b^			% Bias			%(${\hat{\rho }}_{bjj'})\;=\;|1|$		
Method	scenario	*I* ^2^,^a^	${\hat{\beta }}_{1}$	${\hat{\beta }}_{2}$	${\hat{\beta }}_{3}$	${\hat{\beta }}_{1}$	${\hat{\beta }}_{2}$	${\hat{\beta }}_{3}$	*β*_1_	*β*_2_	*β*_3_	${\hat{\tau }}_{1}^{2}$	${\hat{\tau }}_{2}^{2}$	${\hat{\tau }}_{3}^{2}$	${\hat{\tau }}_{1}^{2}$	${\hat{\tau }}_{2}^{2}$	${\hat{\tau }}_{3}^{2}$	${\hat{\rho }}_{b12}$	${\hat{\rho }}_{b13}$	${\hat{\rho }}_{b23}$	${\hat{\rho }}_{b12}$	${\hat{\rho }}_{b13}$	${\hat{\rho }}_{b23}$
MV	COM	50%	0	0	0	0	0	0	96.6	96.1	96.7	11	8	13	-3	-3	-3	-14	-17	-12	16.7	16.9	16.8
UV	COM		0	0	0	0	0	0	95.8	95.3	95.4	3	0	3	0	0	0	—	—	—	—	—	—
MV	COM^c^		0	0	0	0	0	0	96.7	96.1	96.6	11	8	11	-3	-3	-3	-14	-17	-12	16.7	16.8	16.7
MV	MAR		0	0	0	0	1	0	96.7	95.8	96.6	11	18	13	-3	3	0	-22	-18	-20	21.8	21.8	21.9
UV	MAR		0	0	0	0	0	0	95.8	96.6	95.4	3	3	3	0	0	0	—	—	—	—	—	—
MV	MIF		0	9	0	0	-3	0	96.5	86.1	96.4	11	-32	11	-3	-7	0	-29	-17	-28	26.1	25.8	26.2
UV	MIF		0	10	0	0	0	0	95.8	87.2	95.4	3	-47	3	0	0	0	—	—	—	—	—	—
MV	COM	75%	0	0	0	0	0	0	95.2	94.7	95.6	0	1	2	-1	0	-1	-4	-2	-3	2.9	3.5	2.9
UV	COM		0	0	0	0	0	0	95.0	94.7	95.1	-2	-1	0	0	0	0	—	—	—	—	—	—
MV	COM^c^		0	0	0	0	0	0	95.3	94.7	95.5	0	0	2	-1	0	-1	-4	-2	-3	2.7	3.3	2.9
MV	MAR		0	0	0	0	0	0	95.3	93.3	95.6	0	6	2	-1	5	-1	-9	-2	-8	5.1	4.9	5.1
UV	MAR		0	0	0	0	0	0	95.0	94.5	95.1	-2	1	0	0	0	0	—	—	—	—	—	—
MV	MIF		0	13	0	0	-7	0	95.3	78.3	95.7	0	-39	2	-1	1	-1	-18	-2	-19	7.5	6.4	6.8
UV	MIF		0	16	0	0	0	0	95.0	78.1	95.1	-2	-48	0	0	0	0	—	—	—	—	—	—

Abbreviation: RMSE, root mean square error; MV, multivariate meta-analysis; UV, Univariate meta-analysis; COM, complete data scenario; MAR, end point 2 missing at random for 30% studies; MAR, end point 2 missing informatively for 30% studies.

^a^The between-study variances for both end-points $\tau_{j}^{2},\;j\;=\;1,2,3$ are: $\tau_{j}^{2}$ = 0.0036 for *I* ^2^ = 50%, $\tau_{j}^{2}$ = 0.0108 for *I* ^2^ = 75%.

^b^RMSE of estimates by MV method are expressed as % smaller (-) or larger (+) of corresponding estimates by UV method.

^c${\hat{\rho }}_{w}\;$^ ignored.

### Impact of summary data (un)availability

#### Complete data scenario, where within-study correlations, ${\hat{\rho }}_{wi}$’s, were utilized

The percentage of times *ρ*_*b*_’s were estimated at the parameter boundary $\left(i.e.,\;{\hat{\rho }}_{b}\;=\;1\;or\;-1\right)$ were quite high for small meta-analysis (Table 2 and Fig 1), which in general decreased as *m* or *N* or *I* ^2^ increased (Tables 2–5). Also, the relative mean bias and RMSE of ${\hat{\tau }}_{j}^{2}$’s by both approaches decreased as *N* or *m* or *I* ^2^ increased, but those by MV approach become more similar to or smaller than those by UV approach. The mean estimates of effects parameters produced by both approaches were unbiased and very similar (mean bias < .0001 and relative mean bias percentage < 0.1%), where RMSE of ${\hat{\beta }}_{j}$’s were also very similar up to 4 decimal points. In almost all scenarios, there was virtually no difference in the coverage probabilities of the 95% CI by both methods (coverage probability difference < 1%) where both methods almost maintained 0.95 probability.

#### Complete data scenario, where all within-study correlations, ${\hat{\rho }}_{wi}$’s, were missing or ignored

Ignoring ${\hat{\rho }}_{w}$ in MV method when *ρ*_*w*_ ≥ 0.3 resulted in the higher percentage of ${\hat{\rho }}_{b}$ at upper parameter boundary as compared to when ${\hat{\rho }}_{w}$’s were utilized (Tables 2–5). Also, for larger *ρ*_*w*_, ignoring ${\hat{\rho }}_{w}$ resulted in mean ${\hat{\tau }}_{j}^{2}$’s more upwardly biased compared to MV analysis when ${\hat{\rho }}_{w}$’s were available and utilized and compared to UV analysis (Tables 2–5). However, ignoring ${\hat{\rho }}_{w}$’s resulted in no increase of mean bias and RMSE of the effect parameters ${\hat{\beta }}_{j}$’s in MV analysis. Also, there was no or only little impact on the coverage probabilities of *β*_*j*_’s.

#### Missing at random (MAR) scenario, where all ${\hat{\rho }}_{wi}$’s, were utilized

Mean bias on ${\hat{\rho }}_{b}$ was slightly higher and more frequently estimated at the parameter boundary, and mean bias on ${\hat{\tau }}_{j}^{2}$ was slightly higher for non-missing end point and much higher for missing end point in MAR scenario compared to complete data scenarios (Tables 2–5). However, both the UV and MV approaches introduced no or negligible bias in mean ${\hat{\beta }}_{j}’s$ (mean bias ≤ 0.0002, relative mean bias ≤ 0.2%) for both non-missing and missing end points. Also, the RMSE by both approaches were similar for non-missing end point, but those by MV were similar or smaller for randomly missing end point. However, note that the estimates by both methods were quite dispersed (S1 and S4 Figs) resulting in high RMSEs (not shown in tables) for the missing end point. Coverage probabilities of ${\hat{\beta }}_{j}$’s between MV and UV methods were similar for non-missing end point, and almost always maintained 0.95 level. Coverage probability by MV was similar or smaller by about 1~2% than that of UV in general for missing end point.

#### Missing informatively (MIF) scenario, where all ${\hat{\rho }}_{wi}$’s were utilized

The ${\hat{\tau }}_{2}^{2}$ (for missing end point) were often underestimated at low or moderate heterogeneity (*I* ^2^ ≤ 50%) by both approaches for *m* ≥ 10, where UV tended to underestimate more severely and more frequently produced the estimates at the parameter boundary (Tables 2–5, Tables A-J in S2 File, S2 and S5 Figs). For *m* ≥ 15 and *I* ^2^ ≥ 50%, RMSE of ${\hat{\tau }}_{j}^{2}$’s were in general similar or smaller by MV approach. Both the UV and MV methods introduced no or very little bias in ${\hat{\beta }}_{j}’s$ for non-missing end point (relative mean bias ≤ .2%), and also the RMSE of ${\hat{\beta }}_{j}’s$ by two approaches were in general similar. For the missing end point 2, the UV approach that pooled ${\hat{\beta }}_{2i}$’s from significant studies only in general produced similar or greater estimates of *β*_2_ than did MV (where we considered only positive *β*_2_’s in our simulation) in individual replicated data sets (S2 and S5 Figs). The mean bias and RMSE of ${\hat{\beta }}_{2}$ by MV method was almost always smaller, and the difference was much pronounced as *N* or *m* or *I* ^2^ increased (e.g., Table 2 and Table F in S2 File). Coverage probabilities for non-missing end points were similar by both methods; but that for missing end point 2 was much less than 0.95 for UV method while MV method produced much better coverage for m ≥ 10. However, coverage probability of UV approach was in general higher than MV method even for randomly or informatively missing end point for small meta-analysis (i.e., for *m* ≥ 5, where 2 studies were assumed to have missing summary data for end point 2, with UV pooling the end point over just 3 remaining studies) (Tables A and B in S2 File), as expected.

### Impact of varying parameters sizes

#### Varying genetic effects sizes, *β*_j_’s

There was no bias on ${\hat{\beta }}_{j}$’s irrespective of the sizes of the true β_j_’s except in MIF scenarios for missing end point 2, for which the relative mean bias percentage was much higher when the effect sizes were small, e.g., when β_j_ = 0.1, compared to larger effect size, e.g., when $\beta_{j}\approx 0.3$. However, this difference seems to be an artifact of the way relative mean bias is calculated (by dividing the absolute bias by true effect size), where absolute mean biases were very similar in both smaller and larger β_j_’s when other parameters (e.g., *m* or *ρ*_*b*_) were the same.

#### Varying levels of heterogeneity (*I* ^2^’s)

When *I* ^2^ ≥ 50%), MV approach performed similar or better (similar or smaller relative mean bias and RMSE of ${\hat{\beta }}_{2}$ for the missing end point) than univariate approach for MAR and MIF scenarios.

#### Varying meta-analysis size (*m*)

Multivariate approach in general performed similar or better than UV for the estimation of effects parameters when *m* ≥ 10 for *I* ^2^ ≥ 50% and *ρ*_*b*_’s ≥ 0.5 or *ρ*_*w*_’s ≥ 0.5 in MAR and MIF scenarios for missing end point. For *m* = 5, MV approach in general performed similar or worse than UV approach even in high heterogeneity, even in *N* = 10000 or *N* = 20000, and even for missing end point in MAR and MIF scenarios (Tables A and B in S2 File). The coverage probability of UV approach for small *m* was quite high, as expected.

#### Varying within- and between-study correlations (*ρ*_w_’s and *ρ*_b_’s)

Multivariate approach in general performed similar or better than UV for the estimation of effects parameters when *ρ*_*b*_’s ≥ 0.5 or *ρ*_*w*_’s ≥ 0.5 and *m* ≥ 10 at *I* ^2^ ≥ 50% in MAR and MIF scenarios for missing end point.

#### Varying dimension of multivariate analysis (*p* = 2 vs. *p* = 3)

For both *p* = 2 and *p* = 3, the above comparative result seem to hold. However, the estimation of *ρ*_*b*12_, *ρ*_*b*13,_ *ρ*_*b*23_ at the parameter boundary was slightly more frequent for *p* = 3 due to complexities in estimation, where a 3-variate RE meta-analysis requires estimating 6 between-study variance/covariance parameters while a 2-variates requires estimating only 3 such parameters. However, such estimation at the boundary did not seem to impact much on the mean biases and RMSE of the effect parameter estimates and coverage probabilities of the parameters in 3-variate analysis. For example, for *N* = 20000 and *m* = 30 where 3-variate RE meta-analysis requires estimating 6 between-study variance/covariance parameters, multivariate approach seemed performing similarly or better than univariate counterpart in MAR and MIF scenarios with respect to relative mean bias, RMSE and coverage probability for missing end point even when heterogeneity was low (*I* ^2^ ≥ 25%) (Table F in S2 File). This might be because 3-variate meta-analysis can borrow more reliable information for missing end point from two non-missing endpoints. On the other hand, 2-variate analysis does not seem to offer similar degree of advantage in these missing data scenario.

### Impact of unrealistic estimation of nuisance parameters

#### Estimation of *ρ*_*b*_ and $\tau_{j}^{2}$ at the parameters boundaries

Tables 2–5 and Tables A-J in S2 File show how frequently the *ρ*_*b*_ were estimated at the parameter boundary in MV analysis. Fig 1 and S1–S5 Figs show more detail picture of this estimation problem of MV approach in all 5000 replications when true *ρ*_*b*_ = 0.75 in a moderate sized meta-analysis (*m* = 10, *N* = 10000 with an average of 1000 subjects per study) at *p* = 2 and *I* ^2^ = 50%). These figures also show how smaller, similar or larger the ${\hat{\tau }}_{j}^{2}$ and standard errors of ${\hat{\beta }}_{j}$ are by MV compared to UV approach. When ${\hat{\rho }}_{b}\approx \;1$, or ${\hat{\rho }}_{b}\approx \;-1$ (as can be seen in Fig 1 and S1 and S2 Figs for moderate heterogeneity), the MV produced much larger ${\hat{\tau }}_{j}^{2}$ (i.e., ${\hat{\tau }}_{j,\;MV}^{2}/{\hat{\tau }}_{j,\;UV}^{2}>>1$) and consequently larger SE(${\hat{\beta }}_{j}$) (i.e., SE ${\hat{\beta }}_{j,MV})$ / SE(${\hat{\beta }}_{j,UV})>1$) in many replicated data sets. However, note that these large ratios were because UV analysis severely underestimated ${\hat{\tau }}_{j}^{2}$ (including much frequently producing ${\hat{\tau }}_{j,\;UV}^{2}\approx 0$) in those data sets, whereas corresponding ${\hat{\tau }}_{j,\;MV}^{2}$ were much less biased (i.e., biases closer to 0). For larger *m* or weaker *ρ*_*b*_ or greater *I* ^2^, estimation of *ρ*_*b*_ or $\tau_{j}^{2}$ at the parameter boundary were much less frequent. Also note that both the methods tended to either underestimate or overestimate $\tau_{j}^{2}$ in the same direction (i.e., ${\hat{\tau }}_{j}^{2}$ from two approaches were positively correlated) (Fig 1 and S1–S5 Figs).

However, such estimations in the parameter boundaries did not result in higher mean biases or RMSE of pooled estimates in MV than in UV analysis (Tables 2–5 and Tables C-J in S2 File). The average biases on ${\hat{\tau }}_{j}^{2}$’s and ${\hat{\beta }}_{j}$’s were smaller in each of MV and UV analyses among the replications where |${\hat{\rho }}_{b}|<\;1$ or |${\hat{\rho }}_{b}|\leq \;0.95$ than among replications where |${\hat{\rho }}_{b}|\approx \;1$ or |${\hat{\rho }}_{b}|>\;0.95$.

### Performance evaluation using direct sampling of aggregate data

The results of the meta-analysis of sampled aggregate data were consistent with two-stage IPD meta-analysis. Tables G-J in S2 File show that the benefit of multivariate approach over univariate analysis are pronounced for the missing end point in MAR and MIF scenario in high heterogeneity and large *m* and moderate to large within- or between- study correlation. For example, for *p* = 2,*m* = 15,*ρ*_*b*_ = 0.75,*ρ*_*w*_ = 0.75, β_1_ = β_2_ = .1, and $I^{2}\;=\;75\%\;(\tau_{j}^{2}\;=\;.0075)$, and $s_{j}^{2}\;=\;\tau_{j}^{2}/3\;=\;0.0025\;(s_{j}\;=\;.05$ for both *j* = 1,2) under MIF scenario, MV produced much smaller mean bias (${\hat{\beta }}_{2}$ overestimated by 19% in MV vs. 39% in UV analysis) and 25% smaller RMSE of ${\hat{\beta }}_{2}$ for the missing end point (Table J in S2 File). Also, the coverage probability for the corresponding parameter in MV analysis was much better (77.2% in MV vs. 68.2% in UV analysis), although both approaches resulted in lower coverage than nominal level.

## Discussion

We compared the performance of multivariate and univariate approaches to meta-analysis of genetic association studies for the correlated traits via simulation. When all summary data were available from individual studies, MV offered no clear advantage. Also, MV did not offer noticeable advantage even when summary data for some end points were missing randomly (for which MV analysis was seen to offer remarkable benefit [6]) for moderate sized (m = 10) meta-analysis or when there is little variation between studies (*I* ^2^ = 25%). Reason might be that MV requires estimating more parameters (including between-study correlation in between-study variance matrix) simultaneously than univariate one. The estimation of between-study correlation at the parameter boundary was quite often for small or moderate *m* or *I* ^2^, in which univariate approach much severely and frequently underestimated the between study variances (as seen in Fig 1 for *m* = 10), and consequently produced smaller standard errors of the pooled estimates. Also, there were only 3 studies with randomly missing summary data when *m* = 10, which might not be sufficient to produce noticeable benefit of MV over UV approach for such moderate sized meta-analysis. UV analysis offering in general similar or slightly higher coverage for randomly missing end point for small or moderate *m* might be because it relies on fewer available studies, consequently, producing wider confidence interval (as the pooled estimate is expected to be unbiased but both the standard error of pooled estimate and critical value from t-distribution would be larger for UV meta-analysis of fewer studies, even when it usually more severely underestimated between-study variance). However, for larger meta-analysis (*m* ≥ 15) with moderate to large heterogeneity (*I* ^2^ ≥ 50%), such estimation problem were minimal and MV estimates were in general similar or better (i.e., smaller bias and RMSE) for the randomly missing end point.

The biggest advantage of MV method is seen for informatively missing end point for *m* ≥ 15 with *I* ^2^ ≥ 50%, where the relative mean bias, RMSE and the coverage probability for missing end point were better, confirming the previous finding in clinical studies setting [7]. For informatively missing scenario, pooling the summary data from only the significant studies results in upwardly biased pooled estimate when *β*_2_ > 0 (and would be downwardly biased if *β*_2_ < 0 was considered) for the missing end point, a phenomenon known as 'publication bias', in univariate analysis. But, multivariate analysis that assigned null effect for missing summary data (with practically negligible weight for them) might have borrowed the strength of correlation structure to bring otherwise upwardly biased pooled estimate somewhat towards 0, thus decreasing the degree of both mean bias and RMSE, hence somewhat correcting the impact of publication bias. Despite producing wider confidence interval with using fewer studies, UV method might still have lower coverage than MV method for informatively missing end point, perhaps because the pooled estimate in UV analysis was usually much more biased.

A previous study [6] suggested that when between-study correlation are estimated in the parameter boundary (i.e., when ${\hat{\rho }}_{b}\;=\;-1$ or +1), estimates of between-study variances in multivariate approach are generally upwardly biased. We also noted that mean ${\hat{\tau }}_{j}^{2}$’s can (but not necessarily) be upwardly biased when ${\hat{\rho }}_{b}\;=\;+1$ (which was more frequent when *ρ*_*b*_ ≥ 0.5). However, we noticed that ${\hat{\tau }}_{j}^{2}$’s were more frequently downwardly biased (i.e., median ${\hat{\tau }}_{j}^{2}$ downwardly biased) in multivariate analysis when ${\hat{\rho }}_{b}\;=\;+1$ for moderate heterogeneity (e.g., when $\tau_{j}^{2}\;=\;0.0033$ for *I* ^2^ = 50% in complete data scenario as seen in Fig 1). when ${\hat{\rho }}_{b}\;=\;-1$, MV analysis quite frequently underestimated $\tau_{j}^{2}$, where even the mean ${\hat{\tau }}_{j}^{2}$'s were almost always downwardly biased. In such situation, corresponding univariate estimates of $\tau_{j}^{2}$'s were likely to be biased towards the same directions, where UV analysis underestimated $\tau_{j}^{2}$'s much severely and produced the estimates at the parameter boundary more frequently when MV analysis underestimated $\tau_{j}^{2}$'s. Given that univariate approach (that does not condition on ${\hat{\rho }}_{b}$ while estimating between-study variance) tended to underestimate or overestimate between-study variances in the same direction as of multivariate approach, overestimation or underestimation of $\tau_{j}^{2}$'s in MV analysis might not be due to conditioning on ${\hat{\rho }}_{b}\;=\;-1$ or +1. When ${\hat{\tau }}_{j}^{2}$'s are underestimated, the pooled estimates would be more précised in UV analysis, and this might explain why MV analysis that less severely underestimated $\tau_{j}^{2}$'s was unable to produce much better estimates for *m* ≤ 10 or *I* ^2^ = 25% at which ${\hat{\rho }}_{b}\;=\;-1$ or +1 was much frequent.

Despite the complexities of the model and parameters estimation, multivariate approach in general can be useful in moderate to large meta-analysis (*m* ≥ 10, and preferably *m* ≥ 15 studies) with large between-study heterogeneity (*I* ^*2*^ ≥ 50%) and moderate to large correlations (|*ρ*_*w*_| ≥ 0.5 or |*ρ*_*b*_| ≥ 0.5) for an end point with missing summary data in some studies (irrespective of whether it was randomly or informatively missing). However, these results are yet to be seen in real genetic data applications. Also, in real meta-analysis of genetic data, IPD data might not be accessible in one or more studies. Therefore, considering additional data (un)availability scenarios might provide further insights about the performance of these approaches in various real data applications. Comparing these as well as other emerging techniques under univariate and multivariate meta-analysis frameworks in various scenarios mimicking real data applications will be even more helpful for genetic and clinical investigators when they are interested in meta-analyzing two or more correlated end points from genetic association studies.

## Supporting Information

S1 FigBiases in the estimates of *τ*_2_, and biases and SEs of the pooled estimates of *β*_*2*_ from multivariate vs. univariate approaches by whether or not *ρ*_*b*_ is estimated at parameter boundary in 5000 replications in randomly missing summary data scenario^a^.Scenario: $N\;=\;10000,\;m\;=\;10,\;MAF\;=\;0.20,\;\beta_{1}\;=\;0.3,\;\beta_{2}\;=\;0.4,\;\tau_{1}^{2}\;=\;\tau_{2}^{2}\;=\;0.0033;\;I^{2}\;=\;50\%$, *ρ*_*b*_ = 0.75, *ρ*_*w*_ = 0.5. Symbols and abbreviations: *N*, total subjects; *m*, number of studies, *β*_*2*_ and τ_2_, average effect and between-study standard deviation of true study-wise effects for end point 2, respectively; *I* ^*2*^ = degree of between-study heterogeneity; *ρ*_*b*_ and *ρ*_*w*_, true between-and within-study correlations, respectively; MAF, minor allele frequency; SE, standard error; MV, multivariate approach; UV, univariate approach. ^a^Summary data for end point 2 from 3 studies were missing randomly.(TIFF)

S2 FigBiases in the estimates of *τ*_2_, and biases and SEs of the pooled estimates of *β*_*2*_ from multivariate vs. univariate approaches by whether or not *ρ*_*b*_ is estimated at parameter boundary in 5000 replications in informatively missing summary data scenario^a^.Scenario: $N\;=\;10000,\;m\;=\;10,\;MAF\;=\;0.20,\;\beta_{1}\;=\;0.3,\;\beta_{2}\;=\;0.4,\;\tau_{1}^{2}\;=\;\tau_{2}^{2}\;=\;0.0033;\;I^{2}\;=\;50\%$, *ρ*_*b*_ = 0.75, *ρ*_*w*_ = 0.5. Symbols and abbreviations: *N*, total subjects; *m*, number of studies, *β*_*2*_ and τ_2_, average effect and between-study standard deviation of true study-wise effects for end point 2, respectively; *I* ^*2*^ = degree of between-study heterogeneity; *ρ*_*b*_ and *ρ*_*w*_, true between-and within-study correlations, respectively; MAF, minor allele frequency; SE, standard error; MV, multivariate approach; UV, univariate approach. ^a^Summary data for end point 2 from 3 least significant studies were missing (either not reported or unpublished).(TIFF)

S3 FigComparison of the estimates of *β*_*j*_’s, biases in the estimates of *β*_*j*_’s and *τ*_j_’s, and standard errors of estimates of *β*_*j*_’s from multivariate and univariate approaches in 5000 replications in complete summary data scenario.Scenario: $N\;=\;10000,\;m\;=\;10,\;MAF\;=\;0.20,\;\beta_{1}\;=\;0.3,\;\beta_{2}\;=\;0.4,\;\tau_{1}^{2}\;=\;\tau_{2}^{2}\;=\;0.0033;\;I^{2}\;=\;50\%$, *ρ*_*b*_ = 0.75, *ρ*_*w*_ = 0.5. Symbols and abbreviations: *N*, total subjects; *m*, number of studies, *β*_*j*_ and τ_j_, average effect and between-study standard deviation of true study-wise effects for end point *j*, respectively; *I* ^2^ = degree of between-study heterogeneity; *ρ*_*b*_ and *ρ*_*w*_, true between-and within-study correlations, respectively; MAF, minor allele frequency; SE, standard error; MV, multivariate approach; UV, univariate approach.(TIFF)

S4 FigComparison of the estimates of *β*_*j*_’s, biases in the estimates of *β*_*j*_’s and *τ*_j_’s, and standard errors of estimates of *β*_*j*_’s from multivariate and univariate approaches in 5000 replications in randomly missing summary data scenario^a^.Scenario: $N\;=\;10000,\;m\;=\;10,\;MAF\;=\;0.20,\;\beta_{1}\;=\;0.3,\;\beta_{2}\;=\;0.4,\;\tau_{1}^{2}\;=\;\tau_{2}^{2}\;=\;0.0033;\;I^{2}\;=\;50\%$, *ρ*_*b*_ = 0.75, *ρ*_*w*_ = 0.5. Symbols and abbreviations: *N*, total subjects; *m*, number of studies, *β*_*j*_ and *τ*_*j*_, average effect and between-study standard deviation of true study-wise effects for end point *j*, respectively; *I* ^2^ = degree of between-study heterogeneity; *ρ*_*b*_ and *ρ*_*w*_, true between-and within-study correlations, respectively; MAF, minor allele frequency; SE, standard error; MV, multivariate approach; UV, univariate approach. ^a^Summary data for end point 2 from 3 studies were missing randomly.(TIFF)

S5 FigComparison of the estimates of *β*_j_’s, biases in the estimates of *β*_j_’s and *τ*_j_’s, and standard errors of estimates of *β*_j_’s from multivariate and univariate approaches in 5000 replications in informatively missing summary data scenario^a^.Scenario: $N\;=\;10000,\;m\;=\;10,\;MAF\;=\;0.20,\;\beta_{1}\;=\;0.3,\;\beta_{2}\;=\;0.4,\;\tau_{1}^{2}\;=\;\tau_{2}^{2}\;=\;0.0033;\;I^{2}\;=\;50\%$, *ρ*_*b*_ = 0.75, *ρ*_*w*_ = 0.5. Symbols and abbreviations: *N*, total subjects; *m*, number of studies, *β*_*j*_ and *τ*_*j*_, average effect and between-study standard deviation of true study-wise effects for end point *j*, respectively; *I* ^2^ = degree of between-study heterogeneity; *ρ*_*b*_ and *ρ*_*w*_, true between-and within-study correlations, respectively; MAF, minor allele frequency; SE, standard error; MV, multivariate approach; UV, univariate approach. ^a^Summary data for end point 2 from 3 least significant studies were missing (either not reported or unpublished).(TIFF)

S1 File Supplementary Materials and Methods. (DOCX)

S2 File Supplementary Tables. (DOCX)
