# Correction: Multivariate Meta-Analysis of Genetic Association Studies: A Simulation Study

**DOI:** 10.1371/journal.pone.0141520

**Published:** 2015-10-21

**Authors:** The PLOS ONE Staff

## Notice of Republication

This article was republished on October 9, 2015, to correct errors in equations and formatting that were introduced during the typesetting process. The publisher apologizes for the errors. Please download this article again to view the correct version. The originally published, uncorrected article and the republished, corrected article are provided here for reference.

## Supporting Information

S1 File Originally published, uncorrected article. (PDF)

S2 File Republished, corrected article. (PDF)
