# Supplementary figures and images for: Multivariate Meta-Analysis of Genetic Association Studies: A Simulation Study
Source: PLoS One. 2015 Jul 21;10(7):e0133243. doi: 10.1371/journal.pone.0133243 (PMC4509672; doi:10.1371/journal.pone.0133243)

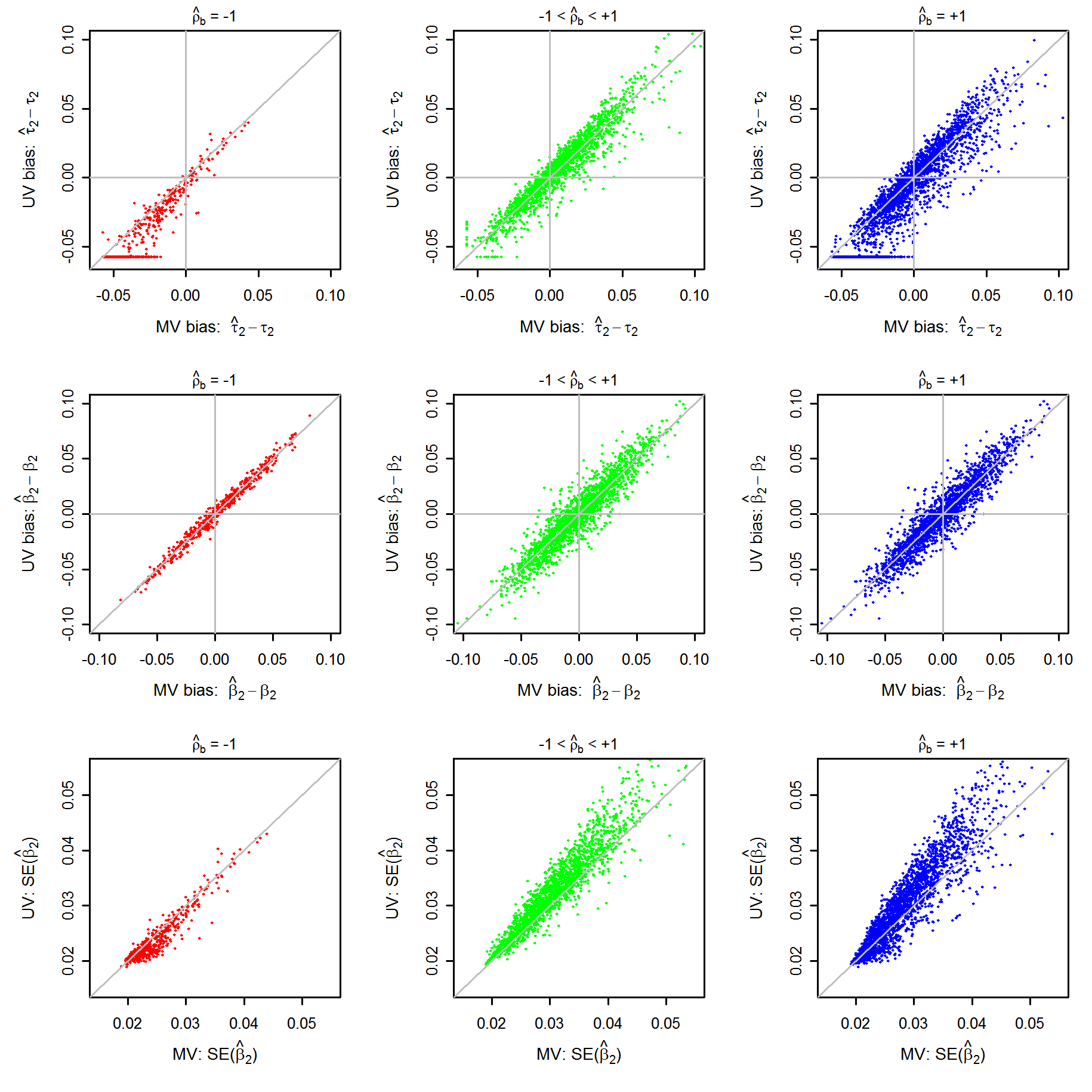

Supplement: S1 Fig — Scenario: N = 10000, m = 10, MAF = 0.20, β1 = 0.3, β2 = 0.4, τ12 = τ22 = 0.0033; I2 = 50%, ρ b = 0.75, ρ w = 0.5. Symbols and abbreviations: N, total subjects; m, number of studies, β 2 and τ2, average effect and between-study standard deviation of true study-wise effects for end point 2, respectively; I 2 = degree of between-study heterogeneity; ρ b and ρ w, true between-and within-study correlations, respectively; MAF, minor allele frequency; SE, standard error; MV, multivariate approach; UV, univariate approach. aSummary data for end point 2 from 3 studies were missing randomly. (TIFF) [file pone.0133243.s001.tiff]

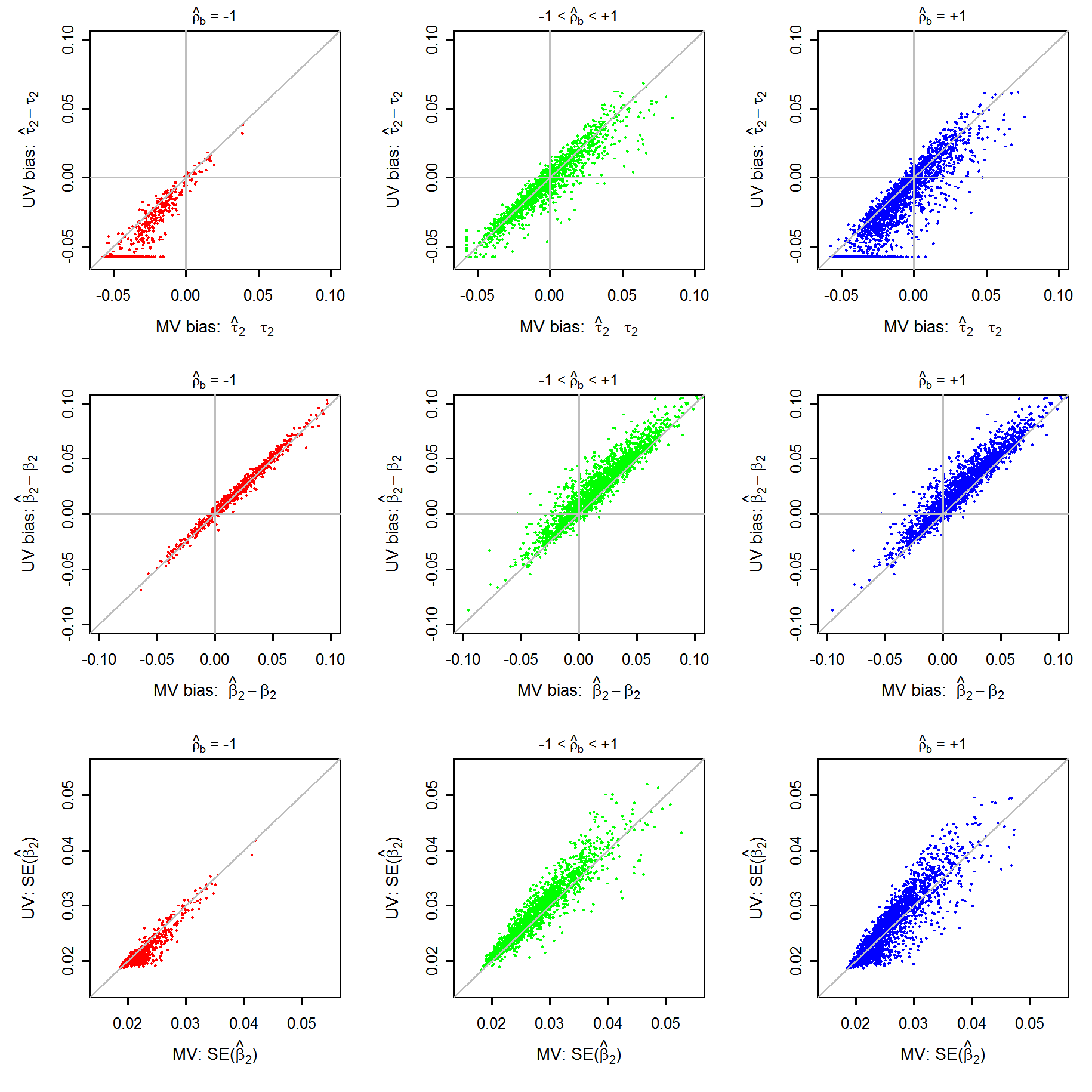

Supplement: S2 Fig — Scenario: N = 10000, m = 10, MAF = 0.20, β1 = 0.3, β2 = 0.4, τ12 = τ22 = 0.0033; I2 = 50%, ρ b = 0.75, ρ w = 0.5. Symbols and abbreviations: N, total subjects; m, number of studies, β 2 and τ2, average effect and between-study standard deviation of true study-wise effects for end point 2, respectively; I 2 = degree of between-study heterogeneity; ρ b and ρ w, true between-and within-study correlations, respectively; MAF, minor allele frequency; SE, standard error; MV, multivariate approach; UV, univariate approach. aSummary data for end point 2 from 3 least significant studies were missing (either not reported or unpublished). (TIFF) [file pone.0133243.s002.tiff]

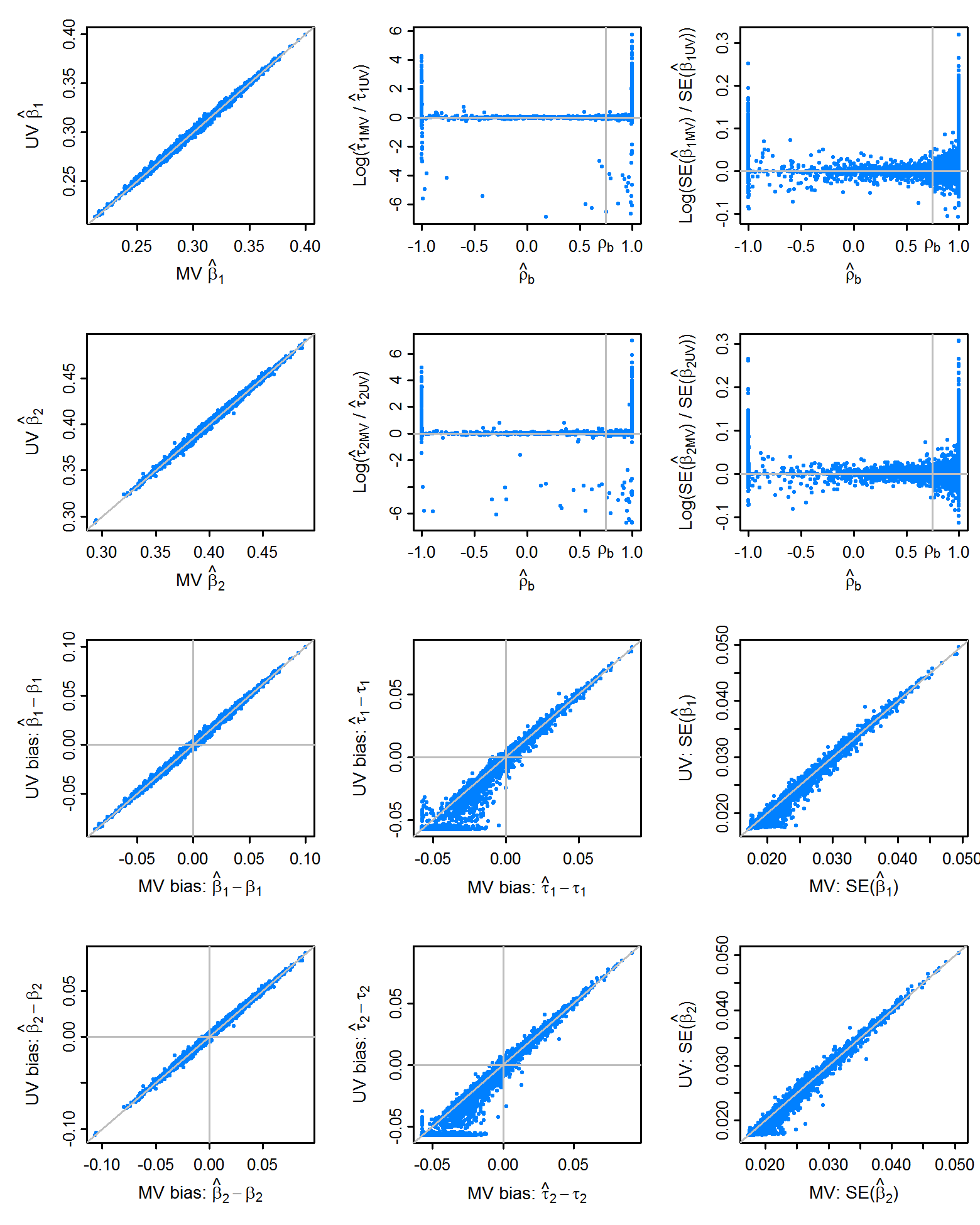

Supplement: S3 Fig — Scenario: N = 10000, m = 10, MAF = 0.20, β1 = 0.3, β2 = 0.4, τ12 = τ22 = 0.0033; I2 = 50%, ρ b = 0.75, ρ w = 0.5. Symbols and abbreviations: N, total subjects; m, number of studies, β j and τj, average effect and between-study standard deviation of true study-wise effects for end point j, respectively; I 2 = degree of between-study heterogeneity; ρ b and ρ w, true between-and within-study correlations, respectively; MAF, minor allele frequency; SE, standard error; MV, multivariate approach; UV, univariate approach. (TIFF) [file pone.0133243.s003.tiff]

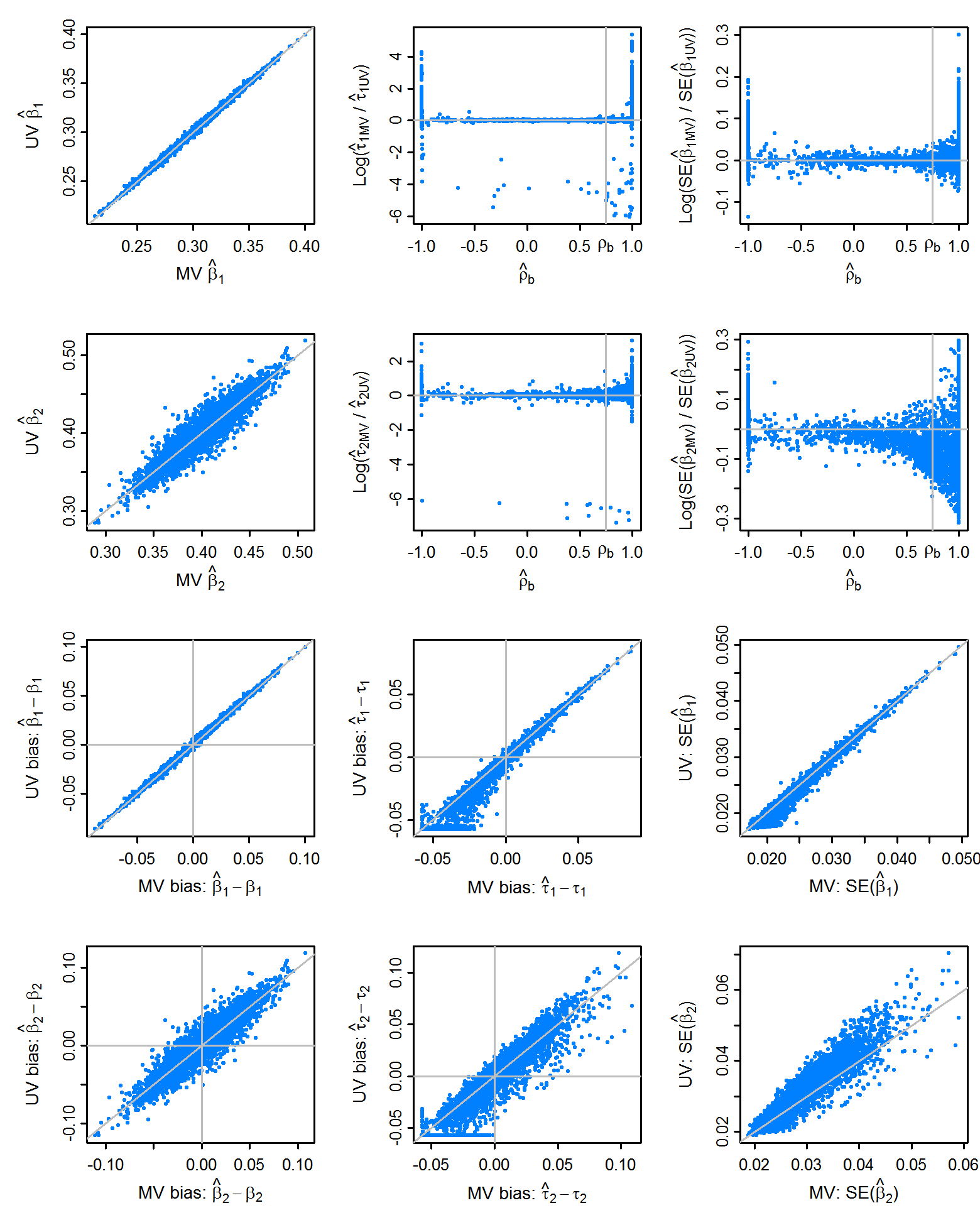

Supplement: S4 Fig — Scenario: N = 10000, m = 10, MAF = 0.20, β1 = 0.3, β2 = 0.4, τ12 = τ22 = 0.0033; I2 = 50%, ρ b = 0.75, ρ w = 0.5. Symbols and abbreviations: N, total subjects; m, number of studies, β j and τ j, average effect and between-study standard deviation of true study-wise effects for end point j, respectively; I 2 = degree of between-study heterogeneity; ρ b and ρ w, true between-and within-study correlations, respectively; MAF, minor allele frequency; SE, standard error; MV, multivariate approach; UV, univariate approach. aSummary data for end point 2 from 3 studies were missing randomly. (TIFF) [file pone.0133243.s004.tiff]

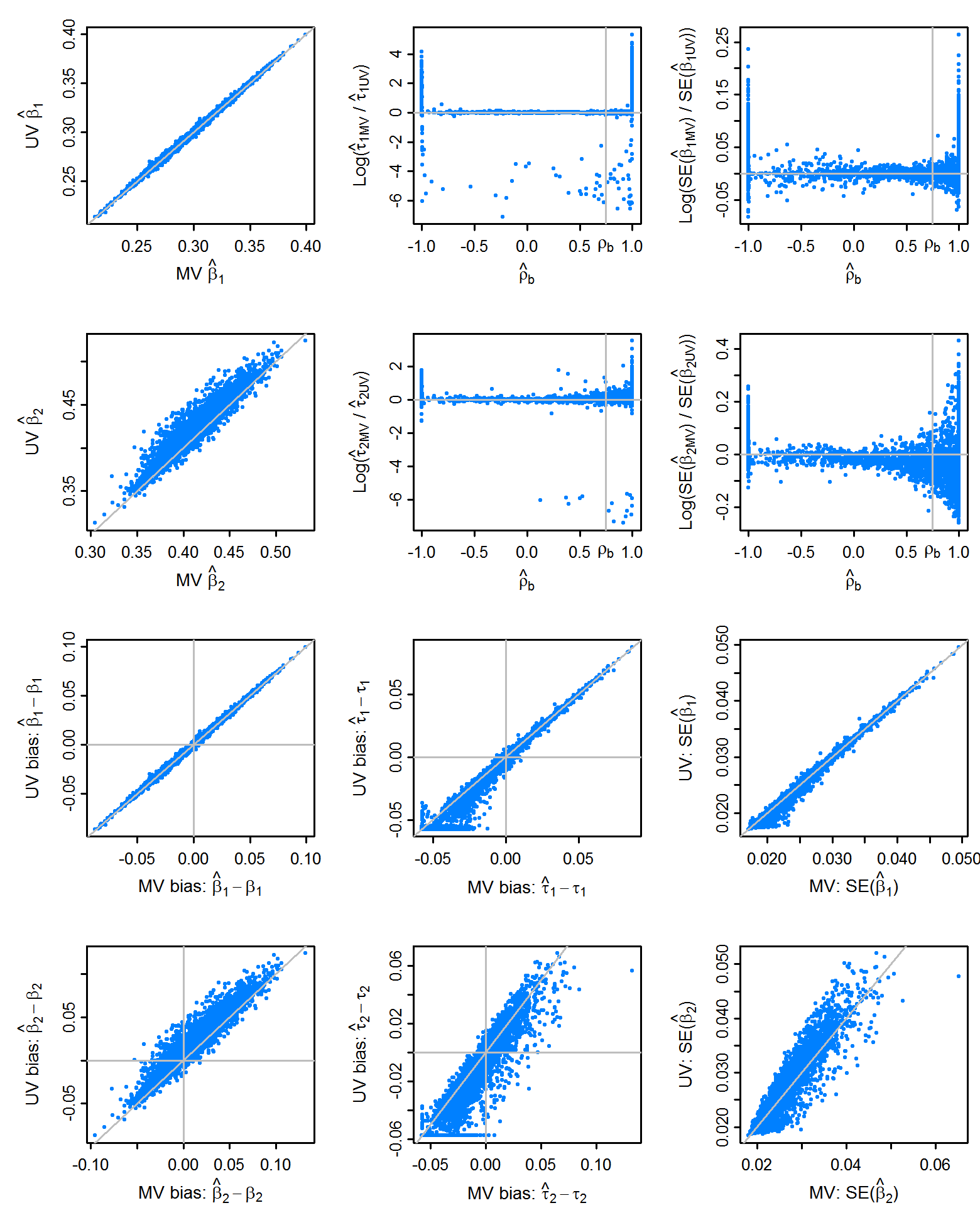

Supplement: S5 Fig — Scenario: N = 10000, m = 10, MAF = 0.20, β1 = 0.3, β2 = 0.4, τ12 = τ22 = 0.0033; I2 = 50%, ρ b = 0.75, ρ w = 0.5. Symbols and abbreviations: N, total subjects; m, number of studies, β j and τ j, average effect and between-study standard deviation of true study-wise effects for end point j, respectively; I 2 = degree of between-study heterogeneity; ρ b and ρ w, true between-and within-study correlations, respectively; MAF, minor allele frequency; SE, standard error; MV, multivariate approach; UV, univariate approach. aSummary data for end point 2 from 3 least significant studies were missing (either not reported or unpublished). (TIFF) [file pone.0133243.s005.tiff]
