# Supplementary material for: Multivariate Meta-Analysis of Genetic Association Studies: A Simulation Study
Source: PLoS One. 2015 Jul 21;10(7):e0133243. doi: 10.1371/journal.pone.0133243 (PMC4509672; doi:10.1371/journal.pone.0133243)
Supplement: S1 File — (DOCX) [file pone.0133243.s006.docx]

# Supplementary Materials and Methods

## A. Adequacy of chosen simulation parameters and accuracy of estimation

As reported in GWAS Catalog [1] (accessed on 1 August 2014) for continuous traits, most of the estimates of genotypic effects on standard deviation (SD) increase in phenotype ranges from $0.05$ to $0.40$ and corresponding standard errors (SEs) (calculated from reported confidence intervals) from $.03\sim0.06$ ($s^{2}=0.0009\sim0.0036$) for many traits when individual study size were around 1000~2000.

For our simulation, we first calculated an approximate value of average within-study variance, $s_{j}^{2}=s^{2}$ (for all $j=1,2...,p$) of the estimate of the effect $\beta_{j}$ of $x$ on the $j$th trait in a study with the average size $n=N/m$ and MAF distribution strictly under HWE for $f=0.20$ (i.e., number of $x=0,1,2$ are $n{(1-f)}^{2}$, $2nf(1-f)$, and $nf^{2}$, respectively, in the study). For this, we generated the data and estimated the variance of $\beta_{j}$ using the model $y_{ijk}^{*}= \alpha+\beta_{j}x+\varepsilon_{ijk}$ ($\varepsilon_{ijk}\sim N(0, \sigma_{\varepsilon}^{2}=1$); $x=0,1,2$) for 1000 times and calculated the average of variances. This variance would roughly be, $Var\left( \hat{\beta}_{j} \right)=s_{j}^{2}=\frac{\sigma_{\varepsilon}^{2}}{nVar\left( x \right)}=\frac{m}{NVar\left( x \right)}$. For example, $s_{j}^{2}\approx0.0036$ was approximated for $m=10$, $N=10000$ and $f=0.20$ (square root $s_{j}$ was first calculated, rounded up to 2 decimal point as $s_{j}\approx0.06,$and then again squared to get rough $s_{j}^{2}$ value)$.$ Then we used it to determine realistic values of $\tau_{j}^{2}$ as $\tau_{j}^{2}=s_{j}^{2}/3, s_{j}^{2}, 3s_{j}^{2}$ for all $j$ for the between-study heterogeneity, $I^{2}=25\%,50\%,$and $75\%$, respectively.

We generated the individual participant data (IPD) using the distribution $\boldsymbol{y}_{ik}^{\boldsymbol{*}}\sim N_{p}\left( \boldsymbol{\alpha}_{i}+\boldsymbol{\beta}_{\boldsymbol{i}}x_{ik}, \boldsymbol{\Psi}_{i} \right)$, where we fixed $\boldsymbol{\alpha}_{i}\boldsymbol{=}(1, 5)^{t}$ for $p= 2$ and $\boldsymbol{\alpha}_{i}\boldsymbol{=}\left( 1, 5,10 \right)^{t}$for $p= 3$ for all $x$ and $m$, and let $\sigma_{\varepsilon_{ij}}^{2}\cong1$ ($\sigma_{\varepsilon_{ij}}^{2}$ being the error variance of $y_{ijk}^{*}$ corresponding to $j$th trait in study $i$) for all $x$ genotypes, $p$ traits and $m$ studies to ensure the identifiability of the model for comparison purpose [2,3,4]. A slightly variable $\sigma_{\varepsilon_{ij}}^{2}$ around 1 would be a closer approximation of real data scenario because the residual variances might not be the same for two or more traits in all $m$ studies. We generated $\sigma_{\varepsilon_{ij}}^{2}$’s from $p$-variate normal with mean $1$’s, SD$=0.05$’s, and $correlation(\sigma_{\varepsilon_{ij}}^{2}, \sigma_{\varepsilon_{ij'}}^{2})=0.75$. For instance, for $p=2$, $(\sigma_{\varepsilon_{i1}}^{2}, \sigma_{\varepsilon_{i2}}^{2})^{t}$ was generated as $\left( \begin{matrix} \sigma_{\varepsilon_{i1}}^{2} \\ \sigma_{\varepsilon_{i2}}^{2} \end{matrix} \right)\sim N_{2}\left( \left( \begin{matrix} 1 \\ 1 \end{matrix} \right), \left( \begin{matrix} {0.05}^{2} & 0.75\times{0.05}^{2} \\ 0.75\times{0.05}^{2} & {0.05}^{2} \end{matrix} \right) \right)$ and then $\boldsymbol{\Psi}_{i}$ was constructed keeping $\rho_{w_{ijj'}}= \rho_{w_{jj'}}$ $j\neq j^{'}=1,2,\ldots,p$ (constant for all $i=1,,\ldots,m$ studies)

as $\boldsymbol{\Psi}_{i}\mathbf{=}\left( \begin{matrix} \sigma_{\varepsilon_{i1}}^{2} & \rho_{w_{12}}\sigma_{\varepsilon_{i1}}\sigma_{\varepsilon_{i2}} \\ \rho_{w_{21}}\sigma_{\varepsilon_{i1}}\sigma_{\varepsilon_{i2}} & \sigma_{\varepsilon_{i2}}^{2} \end{matrix} \right)$.

Once the IPD data were generated in each study, the summary data (namely, $\mathbf{Y}_{i}$ and $\mathbf{S}_{i}$ in study $i$) were obtained by estimating the effect parameters using multivariate linear regression in each study. Following example in a simulation scenario with $p=3$; $\beta_{j}=.3$ for $j=1,2,3$; $m=10$, $N=20000$ (average study size = $2000$), average MAF $=0.20$; $\rho_{b_{ijj'}}=0.5$ (equal) and $\rho_{w_{ijj'}}=0.3$ (equal) between all $j$ and $j’$, and $I^{2}=.75$, gives an idea of the distribution of within study variances and correlation parameters in our simulation: For the 3^rd^ end point, the minimum, 1^st^ quartile, medium, mean, 3^rd^ quartile and maximum values of within-study variance, $s_{3}^{2}$ were $0.0006, 0.0012, 0.0016, 0.0018, 0.0022, 0.0059$ (corresponding $s_{3} = 0.024, 0.035, 0.040, 0.042, 0.047, 0.077$), and that of within-study correlation, for instance, $\hat{\rho}_{w_{32}}$ were, $0.18, 0.29, 0.30, 0.30, 0.31, 0.43,$ respectively, in the 1^st^ replication data set. Here, the estimates of the mean within-study correlation $(\hat{\rho}_{w_{ijj^{'}}})$ were very similar to the assumed $\rho_{w_{ijj'}}$ (i.e., mean$(\hat{\rho}_{w_{ijj^{'}}})\approx\rho_{w_{ijj'}}$ in 3 decimal points) and this was the case in all replications in all scenarios. Further, in each simulated meta-analysis, we calculated average $s_{j}^{2}$ across all $m$ studies using $s_{j}^{2}=(m-1)\sum_{i} w_{ij}/((\sum_{i} w_{ij})^{2}- \sum_{i} w_{ij}^{2})$ where $w_{ij}=1/s_{ij}^{2}$ (assuming large $m$, as in Higgins and Thompson, 2002 [5]). Then, we calculated the estimate of $I^{2}$ for each meta-analysis as $\hat{I}^{2}=\tau_{j}^{2}/(\tau_{j}^{2}+s_{j}^{2})$. The estimated $I^{2}$ in the simulated data sets were very similar to that considered for simulation. For example, $\hat{I}^{2}$ for the 3^rd^ end point ranged from 0.75 to 0.76 in the above scenario.

This confirms the choice of parameters (such as effect sizes, between-study variances and residual variance matrix) was realistic and adequately represented a setting of a meta-analysis of genetic association studies. Also, this suggests that the estimated summary data within studies are realistic and accurate.

## B. Summary of simulation and estimation steps in two-stage IPD meta-analysis

Simulation steps (first stage):

1. Choose a scenario with a combination of a $N$, $m$, $\beta_{j}$’s, $\rho_{w}$(s), $\rho_{b}$(s). Obtain a realistic value of $\tau_{j}^{2}$ for all $j$ at each level of I^2^ and construct $\boldsymbol{\beta}$ and $\boldsymbol{\Sigma}$**.**
2. Simulate $\boldsymbol{\beta}_{\boldsymbol{i}}$ from ${MVN}_{p}\left( \boldsymbol{\beta}, \boldsymbol{\Sigma} \right)$ for study $i$
3. For each$i$, simulate trait values $\boldsymbol{y}_{ik}^{*}$ for subject $k$ from $N_{p}\left( \boldsymbol{\alpha}_{i}+\boldsymbol{\beta}_{\boldsymbol{i}}x, \boldsymbol{\Psi}_{i} \right)$, where
   1. $\boldsymbol{\alpha}_{\boldsymbol{i}}\boldsymbol{=}\left( 1, 5 \right)^{t}$ for $p=2$ and $\boldsymbol{\alpha}_{\boldsymbol{i}}\mathbf{=} \left( 1, 5, 10 \right)^{t}$for $p=3$
   2. $\boldsymbol{\Psi}=\left( \begin{matrix} 1 & \rho_{w} \\ \rho_{w} & 1 \end{matrix} \right)$ for $p=2$and $\boldsymbol{\Psi}=\left( \begin{matrix} 1 & \rho_{w12} & \rho_{w13} \\ \rho_{w21} & 1 & \rho_{w23} \\ \rho_{w31} & \rho_{w32} & 1 \end{matrix} \right)$ for $p=3$
   3. $\boldsymbol{\Psi}_{i}$ was considered little variable around $\boldsymbol{\Psi}$ for different studies where $\rho_{wi}=\rho_{w}.$
4. Estimate ${\mathbf{Y}_{\boldsymbol{i}}=\hat{\boldsymbol{\beta}}}_{i}$ and $\mathbf{S}_{\boldsymbol{i}}$ fitting multivariate linear regression of $\boldsymbol{y}_{ik}^{*}$ on $x$ in study $i$

Estimation steps (second-stage)

1. Meta-analyze by MV and separate UV methods unless otherwise indicated with REML method under RE assumption using data of all end points unless otherwise indicated:
   1. Complete data scenario
   2. Complete data scenario ignoring $\hat{\rho}_{w}$, i.e., setting $\hat{\rho}_{w}=0$ (MV only)
   3. Missing at random scenario: Select 30% studies randomly and set summary data for endpoint 2 missing for those studies.
      1. Discard missing data (for end point 2 only, UV only)
      2. Impute (replace) missing data with $Y_{l2}$= 0, $s_{l2}^{2}$= 10, and $\hat{\rho}_{wl2j}$= 0 for $j\neq2, l=1,2,\ldots m^{'}=0.3m$ (MV only)
   4. Missing informatively scenario: Select 30% studies with smallest $\chi^{2}$ statistics set summary data for endpoint 2 missing for those studies
      1. Discard missing data (for end point 2 only, UV only)
      2. Impute missing data with $Y_{l2}$= 0, $s_{l2}^{2}$= 10, and $\hat{\rho}_{wl2j}$= 0 for $j\neq2, l=1,2,\ldots m^{'}=0.3m$ (MV only)
2. Repeat Steps 1-4 $R=5000$ times (replications).
3. Summarize the estimates over all replications for each parameter where applicable. For example, for effect parameter $\beta_{j}(j=1,\ldots,p)$, calculate
   1. Mean bias ($\hat{\beta}_{j})$ = mean$(\hat{\beta}_{j})-\beta_{j}$
   2. Relative mean bias ($\hat{\beta}_{j})$ percentage (% bias) = ${(\mathrm{mean}(\hat{\beta}}_{j})-\beta_{j})/\beta_{j}\times100\%$
   3. $\mathrm{RMSE}\left( \hat{\beta}_{j} \right)$ =$\sqrt{\sum_{h=1}^{R} {(\hat{\beta}}_{jh}-\beta_{j})^{2}/R}$
   4. Coverage probability ($\beta_{j})$ = #($\beta_{Lower CL,j}\leq\beta_{j}\leq\beta_{Upper CL,j})/R\times100$, where CL is the confidence limit of 95% confidence interval of corresponding parameter.

## C. Method of directly generating aggregate (summary) data

For directly generating (sampling) aggregate (summary) data, we considered 2-variate meta-analysis problem ($p=2$), where chose only one set of effect parameters $\boldsymbol{\beta=(}\beta_{1}=0.1\boldsymbol{,}\beta_{2}{\boldsymbol{=}0.1\boldsymbol{)}}^{t}$**,** two sets of the number of available studies ($m$) as $m=10$ (for $N=10000$), and $m=15$ (for $N=20000$), and two sets of between- and within study correlations $\rho_{b}=0.50$ and $\rho_{w}=0.50$, and $\rho_{b}=0.75$ and $\rho_{w}=0.75$. Thus, we assessed the performances of multivariate and univariate approaches in a total of these 4 scenarios, each at 3 levels of between-study heterogeneity ($I^{2}=25\%, 50\%, 75\%$).

The choice of heterogeneity and within-study parameters was similar to those considered for two-stage IPD meta-analysis. For instance, in a two-stage IPD meta-analysis scenario with average study size $n=N/m=1000$ (e.g., see Table 2 for an IPD data example for $N=10000, m=10$), we used $\tau_{j}^{2}={0.06}^{2}$ for $I^{2}=50\%$. Therefore, for directly generating the aggregate data in the similar $N$ and $m$, we used $s_{j}^{2}={0.06}^{2}=0.0036, and \tau_{j}^{2}={0.06}^{2}/3=0.0012 for I^{2}=25\%, \tau_{j}^{2}={0.06}^{2}=0.0036\mathrm{for}I^{2}=50\%, and \tau_{j}^{2}={3\times0.06}^{2}=0.0108 for I^{2}=75\%$. In another IPD scenario with average study size $N/m=1333$ (e.g., see Table 3 for $=20000,$ $m=15$), we used $\tau_{j}^{2}={0.05}^{2}=0.0025$ for $I^{2}=50\%$. Therefore, we used $s_{j}^{2}={0.05}^{2}=0.0025$, and $\tau_{j}^{2}={0.05}^{2}/3=0.0008$ for $I^{2}=25\%$, $\tau_{j}^{2}={0.05}^{2}=0.0025$ for $I^{2}=50\%$, and $\tau_{j}^{2}={3\times0.05}^{2}=0.0075$ for $I^{2}=75\%$ also for directly generating aggregate data. Thus we constructed between-study variance parameter matrix $\boldsymbol{\Sigma}$ for a scenario.

Also, we needed to generate within-study summary data $\boldsymbol{Y}_{i}$ and $\boldsymbol{S}_{i}$ (i.e., within-study variances ($s_{ij}^{2}$’s), correlations $(\hat{\rho}_{wijj'}$’s), and estimates of effects ($Y_{ij}$’s) in study $i=1,2,\ldots,m$) from some realistic or adequate distributions. Our IPD data examples suggested that the standard deviation of within-study standard error ($s_{ij}$) was SD($s_{ij})\approx0.011$, and the correlation between standard errors for two end points were correlation$\left( s_{ij},s_{ij'} \right)\approx0.98$ on an average in a meta-analysis (replicated) data set for most of the scenarios. Also, the standard deviation of the estimate of within-study correlation between two end points were SD($\hat{\rho}_{wjj'})\approx0.03$ ($j\neq j^{'}=1,\ldots,p$) on an average. We relied on these information to approximate the distributions of within-study standard errors vector and correlation(s), using which we sampled (directly generated) the summary data ($\boldsymbol{Y}_{i}$ and $\boldsymbol{S}_{i}$) in each study. For example, for the scenario with $s_{j}=0.06$ (e.g., $N=10000, m=10$), we generated the study-wise standard error vector $(s_{i1},s_{i2})^{t}$ of (yet-to-be- generated) estimates of effects in individual studies $(Y_{i1},Y_{i2})^{t}$ as $\left( \begin{matrix} s_{i1} \\ s_{i2} \end{matrix} \right)\sim N_{2}\left( \left( \begin{matrix} s_{j} \\ s_{j} \end{matrix} \right), \left( \begin{matrix} {0.011}^{2} & 0.98\times{0.011}^{2} \\ 0.98\times{0.011}^{2} & {0.011}^{2} \end{matrix} \right) \right)$ and $\rho_{wi}$ as $\rho_{wi}\sim N(\rho_{w}, {0.03}^{2})$ in study $i$. The generated $s_{i1}$ and $s_{i2}$ were ensured to be within $\pm2.58\times0.011$ of $s_{j}$ and $\rho_{wi}$ to be within $\pm2.58\times0.03$ of $\rho_{w}$, respectively (covering 99% of the theoretical values under corresponding normal curve). Then $\mathbf{S}_{i}$ was constructed as $\boldsymbol{S}_{i}=\left( \begin{matrix} s_{i1}^{2} & \rho_{wi}s_{i1}s_{i2} \\ \rho_{w}s_{i1}s_{i2} & s_{i2}^{2} \end{matrix} \right)$. Finally, we generated the within study estimates $\boldsymbol{Y}_{i}=(Y_{i1},Y_{i2})^{t}$ were generated using its marginal distribution $\boldsymbol{Y}_{i} \sim N_{p=2}\left( \boldsymbol{\beta}, {\boldsymbol{\Sigma}\boldsymbol{+S}}_{\boldsymbol{i}} \right)$ provided in Equation 6.

The generated summary data for individual studies were then meta-analyzed under multivariate and univariate framework with random-effects assumption under four different data availability scenarios for each of the $R=5000$ replications, and the performance measures were then calculated as in the second stage of IPD meta-analysis ('Estimation steps' 1-3 above).

**References**

1. Hindorff LA MJEBI, Morales J (European Bioinformatics Institute), Junkins HA, Hall PN, Klemm AK, and Manolio TA. A Catalog of Published Genome-Wide Association Studies. Available: [www.genome.gov/gwastudies](http://www.genome.gov/gwastudies). Accessed 1 August 2014.

2. Liu J, Pei Y, Papasian CJ, Deng HW (2009) Bivariate association analyses for the mixture of continuous and binary traits with the use of extended generalized estimating equations. Genetic epidemiology 33: 217-227.

3. Yang F, Tang Z, Deng H (2009) Bivariate association analysis for quantitative traits using generalized estimation equation. Journal of Genetics and Genomics 36: 733-743.

4. Yuan M, Diao G. Joint association analysis of bivariate quantitative and qualitative traits; 2011. BioMed Central Ltd. pp. S74.

5. Higgins J, Thompson SG (2002) Quantifying heterogeneity in a meta‐analysis. Statistics in medicine 21: 1539-1558.
