# Supplementary material for: Multivariate Meta-Analysis of Genetic Association Studies: A Simulation Study
Source: PLoS One. 2015 Jul 21;10(7):e0133243. doi: 10.1371/journal.pone.0133243 (PMC4509672; doi:10.1371/journal.pone.0133243)
Supplement: S2 File — (DOCX) [file pone.0133243.s007.docx]

# Supplementary Tables

**1. Performance evaluations using estimated aggregate data from the first stage of IPD analysis**

**Table A. Relative mean bias percentage, RMSE, and coverage probability when** $\mathbf{N=5000, m=5,}\boldsymbol{\beta}_{\boldsymbol{1}}\boldsymbol{=0.2,}\boldsymbol{\beta}_{\boldsymbol{2}}\boldsymbol{=0.3}$**,** $\boldsymbol{\rho}_{\boldsymbol{b}}$**=0.5,** $\boldsymbol{\rho}_{\boldsymbol{w}}$**=0.**

| Method | Scenario | $I^{2}$,^a^ | Effects | | | | | | Heterogeneity | | | | Correlation | | | |
| --- | --- | --- | --- | --- | --- | --- | --- | --- | --- | --- | --- | --- | --- | --- | --- | --- |
|  |  |  | % Bias | | RMSE^b^ | | Coverage Probability | | % Bias | | RMSE^b^ | | % Bias  ($\hat{\rho}_{b}$) | %($\hat{\rho}_{b})$ | | |
|  |  |  | $\hat{\beta}_{1}$ | $\hat{\beta}_{2}$ | $\hat{\beta}_{1}$ | $\hat{\beta}_{2}$ | $\beta_{1}$ | $\beta_{2}$ | $\hat{\tau}_{1}^{2}$ | $\hat{\tau}_{2}^{2}$ | $\hat{\tau}_{1}^{2}$ | $\hat{\tau}_{2}^{2}$ |  | -1 | +1 | \|1\| |
| MV | COM | 25% | 0 | 0 | 0 | 0 | 98.9 | 99.2 | 69 | 62 | 3 | 4 | -62 | 26.6 | 43.9 | 70.5 |
| UV | COM |  | 0 | 0 | 0 | 0 | 98.7 | 99.0 | 46 | 38 | 0 | 0 | -- | -- | -- | -- |
| MV | COM^c^ |  | 0 | 0 | 0 | 0 | 98.9 | 99.2 | 69 | 62 | 3 | 4 | -63 | 26.9 | 43.6 | 70.5 |
| MV | MAR |  | 0 | 0 | 0 | 3 | 98.8 | 98.8 | 62 | 100 | 3 | 7 | -75 | 30.9 | 42.5 | 73.4 |
| UV | MAR |  | 0 | 0 | 0 | 0 | 98.7 | 100 | 46 | 69 | 0 | 0 | -- | -- | -- | -- |
| MV | MIF |  | 0 | 9 | 0 | 2 | 98.9 | 98.0 | 62 | 23 | 3 | 12 | -75 | 30.6 | 41.9 | 72.5 |
| UV | MIF |  | 0 | 8 | 0 | 0 | 98.7 | 99.9 | 46 | -8 | 0 | 0 | -- | -- | -- | -- |
| MV | COM | 50% | 0 | 0 | 0 | 0 | 97.7 | 98.0 | 16 | 16 | -2 | 0 | -34 | 20.2 | 46.4 | 66.6 |
| UV | COM |  | 0 | 0 | 0 | 0 | 97.2 | 97.5 | 5 | 8 | 0 | 0 | -- | -- | -- | -- |
| MV | COM^c^ |  | 0 | 0 | 0 | 0 | 97.8 | 98.1 | 16 | 16 | -2 | 0 | -34 | 20.1 | 46.4 | 66.5 |
| MV | MAR |  | 0 | 0 | 0 | 5 | 97.8 | 97.2 | 13 | 37 | -2 | 12 | -51 | 28 | 49.7 | 77.7 |
| UV | MAR |  | 0 | 0 | 0 | 0 | 97.2 | 99.8 | 5 | 21 | 0 | 0 | -- | -- | -- | -- |
| MV | MIF |  | 0 | 12 | 0 | 2 | 97.6 | 94.0 | 13 | -21 | -2 | 7 | -57 | 29.4 | 49.1 | 78.5 |
| UV | MIF |  | 0 | 12 | 0 | 0 | 97.2 | 99.4 | 5 | -37 | 0 | 0 | -- | -- | -- | -- |
| MV | COM | 75% | -1 | 0 | 0 | 0 | 96.0 | 96.1 | 4 | 3 | -1 | -2 | -10 | 9.4 | 33 | 42.5 |
| UV | COM |  | -1 | 0 | 0 | 0 | 95.5 | 95.6 | 3 | 1 | 0 | 0 | -- | -- | -- | -- |
| MV | COM^c^ |  | -1 | 0 | 0 | 0 | 96.0 | 96.2 | 4 | 3 | -1 | -2 | -9 | 9.3 | 33.2 | 42.4 |
| MV | MAR |  | -1 | 0 | 0 | 6 | 96.0 | 94.2 | 5 | 18 | -1 | 25 | -29 | 21.4 | 48.7 | 70.1 |
| UV | MAR |  | -1 | 0 | 0 | 0 | 95.5 | 98.9 | 3 | 2 | 0 | 0 | -- | -- | -- | -- |
| MV | MIF |  | -1 | 18 | 0 | 0 | 96.0 | 86.4 | 4 | -30 | -1 | 30 | -44 | 26.0 | 49.1 | 75.1 |
| UV | MIF |  | -1 | 20 | 0 | 0 | 95.5 | 96.6 | 3 | -48 | 0 | 0 | -- | -- | -- | -- |

Abbreviation: RMSE, root mean square error; MV, multivariate meta-analysis; UV, Univariate meta-analysis; COM, complete data scenario; MAR, end point 2 missing at random for 30% studies; MAR, end point 2 missing informatively for 30% studies.

^a^The between-study variances for both end-points $\tau_{j}^{2}, j=1,2$ are: $\tau_{j}^{2}$=0.0013 for $I^{2}$=25, $\tau_{j}^{2}$=0.0038 for $I^{2}$=50, $\tau_{j}^{2}$=0.0114 for $I^{2}$=75.

^b^RMSE of estimates by MV method are expressed as % smaller (-) or larger (+) of corresponding estimates by UV method.

^c^$\hat{\rho}_{w}$ignored.

**Table B. Relative mean bias percentage, RMSE and coverage probability when** $\mathbf{N=10000, m=5,}\boldsymbol{\beta}_{\boldsymbol{1}}\boldsymbol{=0.3,}\boldsymbol{\beta}_{\boldsymbol{2}}\boldsymbol{=0.4}$**,** $\boldsymbol{\rho}_{\boldsymbol{b}}$**=0.75,** $\boldsymbol{\rho}_{\boldsymbol{w}}$**=0.**

| Method | Scenario | $I^{2}$,^a^ | Effects | | | | | | Heterogeneity | | | | Correlation | | | |
| --- | --- | --- | --- | --- | --- | --- | --- | --- | --- | --- | --- | --- | --- | --- | --- | --- |
|  |  |  | % Bias | | RMSE^b^ | | Coverage Probability | | % Bias | | RMSE^b^ | | % Bias  ($\hat{\rho}_{b}$) | %($\hat{\rho}_{b})$ | | |
|  |  |  | $\hat{\beta}_{1}$ | $\hat{\beta}_{2}$ | $\hat{\beta}_{1}$ | $\hat{\beta}_{2}$ | $\beta_{1}$ | $\beta_{2}$ | $\hat{\tau}_{1}^{2}$ | $\hat{\tau}_{2}^{2}$ | $\hat{\tau}_{1}^{2}$ | $\hat{\tau}_{2}^{2}$ |  | -1 | +1 | \|1\| |
| MV | COM | 25% | 0 | 0 | 0 | 0 | 99.4 | 99.3 | 67 | 67 | 8 | 7 | -68 | 24.8 | 45.5 | 70.3 |
| UV | COM |  | 0 | 0 | 0 | 0 | 99.1 | 99.1 | 50 | 50 | 0 | 0 | -- | -- | -- | -- |
| MV | COM^c^ |  | 0 | 0 | 0 | 0 | 99.4 | 99.3 | 67 | 67 | 8 | 7 | -69 | 24.7 | 45.5 | 70.2 |
| MV | MAR |  | 0 | 0 | 0 | 3 | 99.3 | 99.0 | 67 | 117 | 8 | 5 | -79 | 29.9 | 44.3 | 74.2 |
| UV | MAR |  | 0 | 0 | 0 | 0 | 99.1 | 100 | 50 | 83 | 0 | 0 | -- | -- | -- | -- |
| MV | MIF |  | 0 | 3 | 0 | 1 | 99.3 | 98.7 | 67 | 67 | 8 | 7 | -75 | 28.2 | 45.3 | 73.6 |
| UV | MIF |  | 0 | 3 | 0 | 0 | 99.1 | 100 | 50 | 33 | 0 | 0 | -- | -- | -- | -- |
| MV | COM | 50% | 0 | 0 | 0 | 0 | 97.9 | 97.6 | 21 | 16 | 0 | 0 | -34 | 14.5 | 54.3 | 68.9 |
| UV | COM |  | 0 | 0 | 0 | 0 | 97.2 | 97.2 | 11 | 11 | 0 | 0 | -- | -- | -- | -- |
| MV | COM^c^ |  | 0 | 0 | 0 | 0 | 97.9 | 97.6 | 21 | 16 | 0 | 0 | -34 | 14.8 | 54.5 | 69.3 |
| MV | MAR |  | 0 | 0 | 0 | 3 | 97.6 | 97.5 | 16 | 37 | 0 | 9 | -50 | 22.7 | 57.3 | 80 |
| UV | MAR |  | 0 | 0 | 0 | 0 | 97.2 | 99.9 | 11 | 21 | 0 | 0 | -- | -- | -- | -- |
| MV | MIF |  | 0 | 4 | 0 | 1 | 97.6 | 95.8 | 16 | 5 | 0 | 15 | -53 | 23.5 | 55.2 | 78.7 |
| UV | MIF |  | 0 | 4 | 0 | 0 | 97.2 | 99.8 | 11 | -16 | 0 | 0 | -- | -- | -- | -- |
| MV | COM | 75% | 0 | 0 | 0 | 0 | 96.2 | 96.3 | 5 | 5 | 0 | 0 | -9 | 5.6 | 48.0 | 53.7 |
| UV | COM |  | 0 | 0 | 0 | 0 | 95.5 | 95.6 | 2 | 2 | 0 | 0 | -- | -- | -- | -- |
| MV | COM^c^ |  | 0 | 0 | 0 | 0 | 96.2 | 96.3 | 5 | 5 | 0 | 0 | -10 | 5.6 | 48 | 53.6 |
| MV | MAR |  | 0 | 0 | 0 | 2 | 96.2 | 94.2 | 5 | 14 | 0 | 12 | -27 | 15.1 | 60.4 | 75.5 |
| UV | MAR |  | 0 | 0 | 0 | 0 | 95.5 | 98.5 | 2 | 4 | 0 | 0 | -- | -- | -- | -- |
| MV | MIF |  | 0 | 7 | 0 | -2 | 96.1 | 89.9 | 5 | -11 | 0 | 34 | -35 | 17.4 | 58.4 | 75.8 |
| UV | MIF |  | 0 | 8 | 0 | 0 | 95.5 | 97.0 | 2 | -32 | 0 | 0 | -- | -- | -- | -- |

Abbreviation: RMSE, root mean square error; MV, multivariate meta-analysis; UV, Univariate meta-analysis; COM, complete data scenario; MAR, end point 2 missing at random for 30% studies; MAR, end point 2 missing informatively for 30% studies.

^a^The between-study variances for both end-points $\tau_{j}^{2}, j=1,2$ are: $\tau_{j}^{2}$=0.0006 for $I^{2}$=25, $\tau_{j}^{2}$=0.0019 for $I^{2}$=50, $\tau_{j}^{2}$=0.0057 for $I^{2}$=75.

^b^RMSE of estimates by MV method are expressed as % smaller (-) or larger (+) of corresponding estimates by UV method.

^c^$\hat{\rho}_{w}$ignored.

**Table C. Relative mean bias percentage, RMSE and coverage probability when** $\mathbf{N=20000, m=20,}\boldsymbol{\beta}_{\boldsymbol{1}}\boldsymbol{=0.1,}\boldsymbol{\beta}_{\boldsymbol{2}}\boldsymbol{=0.1}$**,** $\boldsymbol{\rho}_{\boldsymbol{b}}$**=0.6,** $\boldsymbol{\rho}_{\boldsymbol{w}}$**=0.3.**

| Method | Scenario | $I^{2}$,^a^ | Effects | | | | | | Heterogeneity | | | | Correlation | | | |
| --- | --- | --- | --- | --- | --- | --- | --- | --- | --- | --- | --- | --- | --- | --- | --- | --- |
|  |  |  | % Bias | | RMSE^b^ | | Coverage Probability | | % Bias | | RMSE^b^ | | % Bias  ($\hat{\rho}_{b}$) | %($\hat{\rho}_{b})$ | | |
|  |  |  | $\hat{\beta}_{1}$ | $\hat{\beta}_{2}$ | $\hat{\beta}_{1}$ | $\hat{\beta}_{2}$ | $\beta_{1}$ | $\beta_{2}$ | $\hat{\tau}_{1}^{2}$ | $\hat{\tau}_{2}^{2}$ | $\hat{\tau}_{1}^{2}$ | $\hat{\tau}_{2}^{2}$ |  | -1 | +1 | \|1\| |
| MV | COM | 25% | 0 | 0 | 0 | 0 | 95.9 | 95.8 | 17 | 17 | 0 | 0 | -24 | 13.8 | 42.8 | 56.5 |
| UV | COM |  | 0 | 0 | 0 | 0 | 95.6 | 95.4 | 8 | 8 | 0 | 0 | -- | -- | -- | -- |
| MV | COM^c^ |  | 0 | 0 | 0 | 0 | 96.1 | 96.0 | 33 | 33 | 0 | 0 | 41 | 2.7 | 76 | 78.7 |
| MV | MAR |  | 0 | 0 | 0 | -2 | 95.8 | 95.5 | 17 | 25 | 0 | 0 | -33 | 16.8 | 44.8 | 61.6 |
| UV | MAR |  | 0 | 0 | 0 | 0 | 95.6 | 95.8 | 8 | 17 | 0 | 0 | -- | -- | -- | -- |
| MV | MIF |  | 0 | 24 | 0 | -7 | 95.8 | 68.7 | 17 | -50 | 0 | -8 | -50 | 25.9 | 48.3 | 74.2 |
| UV | MIF |  | 0 | 27 | 0 | 0 | 95.6 | 64.7 | 8 | -58 | 0 | 0 | -- | -- | -- | -- |
| MV | COM | 50% | 0 | 0 | 1 | 0 | 95.4 | 94.8 | 3 | 0 | 0 | 0 | 1 | 2.1 | 20.5 | 22.6 |
| UV | COM |  | 0 | 0 | 0 | 0 | 95.3 | 94.7 | 0 | 0 | 0 | 0 | -- | -- | -- | -- |
| MV | COM^c^ |  | 0 | 0 | 1 | 0 | 95.5 | 95.2 | 6 | 6 | 0 | 0 | 39 | 0.5 | 48.9 | 49.4 |
| MV | MAR |  | 0 | 0 | 1 | -2 | 95.3 | 94.6 | 3 | 3 | 0 | -4 | -2 | 4.3 | 28.5 | 32.8 |
| UV | MAR |  | 0 | 0 | 0 | 0 | 95.3 | 95.2 | 0 | 0 | 0 | 0 | -- | -- | -- | -- |
| MV | MIF |  | 0 | 26 | 0 | -10 | 95.4 | 66.9 | 3 | -28 | 0 | -3 | -14 | 10.4 | 37.2 | 47.6 |
| UV | MIF |  | 0 | 31 | 0 | 0 | 95.3 | 62.5 | 0 | -33 | 0 | 0 | -- | -- | -- | -- |
| MV | COM | 75% | 0 | 0 | 0 | 0 | 95.6 | 95.0 | 1 | 1 | 0 | 0 | -1 | 0 | 1.2 | 1.2 |
| UV | COM |  | 0 | 0 | 0 | 0 | 95.7 | 95.0 | 1 | 1 | 0 | 0 | -- | -- | -- | -- |
| MV | COM^c^ |  | 0 | 0 | 0 | 0 | 95.6 | 95.0 | 2 | 1 | 0 | 0 | 16 | 0 | 5.9 | 5.9 |
| MV | MAR |  | 0 | 0 | 0 | -3 | 95.6 | 94.1 | 1 | 2 | 0 | 2 | 0 | 0.2 | 4.3 | 4.5 |
| UV | MAR |  | 0 | 0 | 0 | 0 | 95.7 | 95.3 | 1 | 1 | 0 | 0 | -- | -- | -- | -- |
| MV | MIF |  | 0 | 26 | 0 | -13 | 95.7 | 79.8 | 1 | 6 | 0 | -1 | -2 | 0.7 | 5.1 | 5.7 |
| UV | MIF |  | 0 | 36 | 0 | 0 | 95.7 | 76.7 | 1 | 6 | 0 | 0 | -- | -- | -- | -- |

Abbreviation: RMSE, root mean square error; MV, multivariate meta-analysis; UV, Univariate meta-analysis; COM, complete data scenario; MAR, end point 2 missing at random for 30% studies; MAR, end point 2 missing informatively for 30% studies.

^a^The between-study variances for both end-points $\tau_{j}^{2}, j=1,2$ are: $\tau_{j}^{2}$=0.0012 for $I^{2}$=25, $\tau_{j}^{2}$=0.0036 for $I^{2}$=50, $\tau_{j}^{2}$=0.0108 for $I^{2}$=75.

^b^RMSE of estimates by MV method are expressed as % smaller (-) or larger (+) of corresponding estimates by UV method.

^c^$\hat{\rho}_{w}$ignored.

**Table D. Relative mean bias percentage, RMSE and coverage probability when** ${\mathbf{N=20000, m=15,}\boldsymbol{\beta}}_{\boldsymbol{1}}\boldsymbol{=0.2,}\boldsymbol{\beta}_{\boldsymbol{2}}\boldsymbol{=0.3}$**,** $\boldsymbol{\rho}_{\boldsymbol{b}}$**=0.6,** $\boldsymbol{\rho}_{\boldsymbol{w}}$**=0.3.**

| Method | Scenario | $I^{2}$,^a^ | Effects | | | | | | Heterogeneity | | | | Correlation | | | |
| --- | --- | --- | --- | --- | --- | --- | --- | --- | --- | --- | --- | --- | --- | --- | --- | --- |
|  |  |  | % Bias | | RMSE^b^ | | Coverage Probability | | % Bias | | RMSE^b^ | | % Bias  ($\hat{\rho}_{b}$) | %($\hat{\rho}_{b})$ | | |
|  |  |  | $\hat{\beta}_{1}$ | $\hat{\beta}_{2}$ | $\hat{\beta}_{1}$ | $\hat{\beta}_{2}$ | $\beta_{1}$ | $\beta_{2}$ | $\hat{\tau}_{1}^{2}$ | $\hat{\tau}_{2}^{2}$ | $\hat{\tau}_{1}^{2}$ | $\hat{\tau}_{2}^{2}$ |  | -1 | +1 | \|1\| |
| MV | COM | 25% | 0 | 0 | 0 | 1 | 95.9 | 96.8 | 22 | 22 | 0 | 0 | -38 | 18 | 43.2 | 61.2 |
| UV | COM |  | 0 | 0 | 0 | 0 | 95.6 | 96.3 | 11 | 11 | 0 | 0 | -- | -- | -- | -- |
| MV | COM^c^ |  | 0 | 0 | 0 | 0 | 96.4 | 97.0 | 33 | 44 | 0 | 9 | 27 | 5.4 | 71.3 | 76.7 |
| MV | MAR |  | 0 | 0 | 0 | -2 | 96.0 | 96.2 | 22 | 33 | 0 | 0 | -41 | 20.9 | 46.4 | 67.3 |
| UV | MAR |  | 0 | 0 | 0 | 0 | 95.6 | 96.8 | 11 | 22 | 0 | 0 | -- | -- | -- | -- |
| MV | MIF |  | 0 | 5 | 0 | -5 | 95.8 | 87.6 | 22 | -22 | 0 | 0 | -52 | 24.4 | 45.6 | 70 |
| UV | MIF |  | 0 | 6 | 0 | 0 | 95.6 | 87.5 | 11 | -33 | 0 | 0 | -- | -- | -- | -- |
| MV | COM | 50% | 0 | 0 | 1 | 0 | 95.1 | 95.6 | 7 | 4 | -5 | -5 | -5 | 4.8 | 24.9 | 29.7 |
| UV | COM |  | 0 | 0 | 0 | 0 | 94.7 | 95.3 | 4 | 4 | 0 | 0 | -- | -- | -- | -- |
| MV | COM^c^ |  | 0 | 0 | 1 | 0 | 95.2 | 95.9 | 11 | 7 | -5 | -5 | 33 | 1.4 | 50.6 | 52 |
| MV | MAR |  | 0 | 0 | 1 | -2 | 95.1 | 94.5 | 7 | 7 | -5 | 0 | -11 | 8.2 | 34.6 | 42.8 |
| UV | MAR |  | 0 | 0 | 0 | 0 | 94.7 | 95.3 | 4 | 4 | 0 | 0 | -- | -- | -- | -- |
| MV | MIF |  | 0 | 7 | 1 | -10 | 95.0 | 80.7 | 7 | -41 | -5 | 0 | -26 | 12.2 | 36.2 | 48.4 |
| UV | MIF |  | 0 | 8 | 0 | 0 | 94.7 | 79.0 | 4 | -44 | 0 | 0 | -- | -- | -- | -- |
| MV | COM | 75% | 0 | 0 | 0 | 0 | 94.8 | 95.3 | -1 | 0 | 0 | 0 | -2 | 0.1 | 3.6 | 3.7 |
| UV | COM |  | 0 | 0 | 0 | 0 | 95.0 | 95.3 | -1 | 0 | 0 | 0 | -- | -- | -- | -- |
| MV | COM^c^ |  | 0 | 0 | 0 | 0 | 94.9 | 95.2 | 0 | 1 | 0 | 0 | 16 | 0.1 | 10.8 | 10.9 |
| MV | MAR |  | 0 | 0 | 0 | -3 | 95.0 | 93.6 | -1 | 1 | 0 | 2 | -4 | 0.9 | 9.8 | 10.7 |
| UV | MAR |  | 0 | 0 | 0 | 0 | 95.0 | 95.1 | -1 | 0 | 0 | 0 | -- | -- | -- | -- |
| MV | MIF |  | 0 | 12 | 0 | -13 | 94.9 | 70.3 | -1 | -44 | 0 | 0 | -20 | 2.9 | 12.5 | 15.5 |
| UV | MIF |  | 0 | 15 | 0 | 0 | 95.0 | 66.0 | -1 | -49 | 0 | 0 | -- | -- | -- | -- |

Abbreviation: RMSE, root mean square error; MV, multivariate meta-analysis; UV, Univariate meta-analysis; COM, complete data scenario; MAR, end point 2 missing at random for 30% studies; MAR, end point 2 missing informatively for 30% studies.

^a^The between-study variances for both end-points $\tau_{j}^{2}, j=1,2$ are: $\tau_{j}^{2}$=0.0009 for $I^{2}$=25, $\tau_{j}^{2}$=0.0027 for $I^{2}$=50, $\tau_{j}^{2}$=0.0082 for $I^{2}$=75.

^b^RMSE of estimates by MV method are expressed as % smaller (-) or larger (+) of corresponding estimates by UV method.

^c^$\hat{\rho}_{w}$ignored.

**Table E. Relative mean bias percentage, RMSE and coverage probability when** $\mathbf{N=30000, m=30,}\boldsymbol{\beta}_{\boldsymbol{1}}\boldsymbol{=0.1,}\boldsymbol{\beta}_{\boldsymbol{2}}\boldsymbol{=0.1}$**,** $\boldsymbol{\rho}_{\boldsymbol{b}}$**=0.6,** $\boldsymbol{\rho}_{\boldsymbol{w}}$**=0.3.**

| Method | Scenario | $I^{2}$,^a^ | Effects | | | | | | Heterogeneity | | | | Correlation | | | |
| --- | --- | --- | --- | --- | --- | --- | --- | --- | --- | --- | --- | --- | --- | --- | --- | --- |
|  |  |  | % Bias | | RMSE^b^ | | Coverage Probability | | % Bias | | RMSE^b^ | | % Bias  ($\hat{\rho}_{b}$) | %($\hat{\rho}_{b})$ | | |
|  |  |  | $\hat{\beta}_{1}$ | $\hat{\beta}_{2}$ | $\hat{\beta}_{1}$ | $\hat{\beta}_{2}$ | $\beta_{1}$ | $\beta_{2}$ | $\hat{\tau}_{1}^{2}$ | $\hat{\tau}_{2}^{2}$ | $\hat{\tau}_{1}^{2}$ | $\hat{\tau}_{2}^{2}$ |  | -1 | +1 | \|1\| |
| MV | COM | 25% | 0 | 0 | 0 | 0 | 95.8 | 95.7 | 8 | 8 | -9 | -9 | -16 | 10.1 | 37.7 | 47.8 |
| UV | COM |  | 0 | 0 | 0 | 0 | 95.7 | 95.5 | 8 | 8 | 0 | 0 | -- | -- | -- | -- |
| MV | COM^c^ |  | 0 | 0 | 0 | 0 | 96.4 | 96.2 | 33 | 33 | 0 | 0 | 51 | 1 | 78.7 | 79.7 |
| MV | MAR |  | 0 | 0 | 0 | -2 | 95.9 | 95.2 | 8 | 17 | -9 | -8 | -21 | 12.6 | 42.6 | 55.2 |
| UV | MAR |  | 0 | 0 | 0 | 0 | 95.7 | 95.5 | 8 | 8 | 0 | 0 | -- | -- | -- | -- |
| MV | MIF |  | 0 | 24 | 0 | -8 | 95.8 | 53.5 | 8 | -58 | -9 | 0 | -48 | 25.2 | 49.5 | 74.7 |
| UV | MIF |  | 0 | 27 | 0 | 0 | 95.7 | 47.4 | 8 | -67 | 0 | 0 | -- | -- | -- | -- |
| MV | COM | 50% | 0 | 0 | 0 | 0 | 95.1 | 95.2 | 0 | 3 | 0 | 0 | 1 | 0.7 | 9.4 | 10.1 |
| UV | COM |  | 0 | 0 | 0 | 0 | 95.1 | 95.3 | 0 | 3 | 0 | 0 | -- | -- | -- | -- |
| MV | COM^c^ |  | 0 | 0 | 0 | 0 | 95.3 | 95.4 | 6 | 6 | 0 | 0 | 40 | 0.1 | 39.5 | 39.6 |
| MV | MAR |  | 0 | 0 | 0 | -2 | 95.1 | 94.8 | 0 | 3 | 0 | 0 | -2 | 1.9 | 16.3 | 18.2 |
| UV | MAR |  | 0 | 0 | 0 | 0 | 95.1 | 95.3 | 0 | 3 | 0 | 0 | -- | -- | -- | -- |
| MV | MIF |  | 0 | 27 | 0 | -12 | 95.2 | 57.8 | 0 | -31 | 0 | -4 | -9 | 6.4 | 26.8 | 33.2 |
| UV | MIF |  | 0 | 32 | 0 | 0 | 95.1 | 49.7 | 0 | -33 | 0 | 0 | -- | -- | -- | -- |
| MV | COM | 75% | 0 | 0 | 0 | 0 | 94.8 | 94.5 | 1 | 0 | 0 | 0 | -2 | 0 | 0.1 | 0.1 |
| UV | COM |  | 0 | 0 | 0 | 0 | 94.9 | 94.4 | 1 | 0 | 0 | 0 | -- | -- | -- | -- |
| MV | COM^c^ |  | 0 | 0 | 0 | 0 | 94.9 | 94.4 | 1 | 1 | 0 | 0 | 15 | 0 | 1.7 | 1.7 |
| MV | MAR |  | 0 | 0 | 0 | -3 | 94.8 | 94.1 | 1 | 1 | 0 | 2 | -2 | 0 | 0.7 | 0.7 |
| UV | MAR |  | 0 | 0 | 0 | 0 | 94.9 | 94.5 | 1 | 1 | 0 | 0 | -- | -- | -- | -- |
| MV | MIF |  | 0 | 26 | 0 | -16 | 94.8 | 76.1 | 1 | 6 | 0 | -4 | -1 | 0.1 | 1 | 1.1 |
| UV | MIF |  | 0 | 36 | 0 | 0 | 94.9 | 69.9 | 1 | 6 | 0 | 0 | -- | -- | -- | -- |

Abbreviation: RMSE, root mean square error; MV, multivariate meta-analysis; UV, Univariate meta-analysis; COM, complete data scenario; MAR, end point 2 missing at random for 30% studies; MAR, end point 2 missing informatively for 30% studies.

^a^The between-study variances for both end-points $\tau_{j}^{2}, j=1,2$ are: $\tau_{j}^{2}$=0.0012 for $I^{2}$=25, $\tau_{j}^{2}$=0.0036 for $I^{2}$=50, $\tau_{j}^{2}$=0.0108 for $I^{2}$=75.

^b^RMSE of estimates by MV method are expressed as % smaller (-) or larger (+) of corresponding estimates by UV method.

^c^$\hat{\rho}_{w}$ignored.

**Table F. Relative mean bias percentage, RMSE and coverage probability when** $\boldsymbol{p=3,}\mathbf{N=20000, m=30,}\boldsymbol{\beta}_{\boldsymbol{1}}\boldsymbol{=}\boldsymbol{\beta}_{\boldsymbol{2}}\boldsymbol{=}\boldsymbol{\beta}_{\boldsymbol{3}}\boldsymbol{=0.1}$**,** $\boldsymbol{\rho}_{\boldsymbol{b}\boldsymbol{12}}\mathbf{=0.6}$**,** $\boldsymbol{\rho}_{\boldsymbol{b}\boldsymbol{13}}\mathbf{=0.5}$**,** $\boldsymbol{\rho}_{\boldsymbol{b}\boldsymbol{23}}\mathbf{=0.7}$**,** $\boldsymbol{\rho}_{\boldsymbol{w}\boldsymbol{12}}\mathbf{=}\boldsymbol{\rho}_{\boldsymbol{w}\boldsymbol{13}}\mathbf{=}\boldsymbol{\rho}_{\boldsymbol{w}\boldsymbol{23}}\mathbf{=0.3}$**.**

| Method | Scenario | $I^{2}$,^a^ | Effects | | | | | | | | | Heterogeneity | | | | | | Correlation | | | | | |
| --- | --- | --- | --- | --- | --- | --- | --- | --- | --- | --- | --- | --- | --- | --- | --- | --- | --- | --- | --- | --- | --- | --- | --- |
|  |  |  | % Bias | | | RMSE^b^ | | | Coverage Prob | | | % Bias | | | RMSE^b^ | | | % Bias | | | %($\hat{\rho}_{bjj'})=\vert1\vert$ | | |
|  |  |  | $\hat{\beta}_{1}$ | $\hat{\beta}_{2}$ | $\hat{\beta}_{3}$ | $\hat{\beta}_{1}$ | $\hat{\beta}_{2}$ | $\hat{\beta}_{3}$ | $\beta_{1}$ | $\beta_{2}$ | $\beta_{3}$ | $\hat{\tau}_{1}^{2}$ | $\hat{\tau}_{2}^{2}$ | $\hat{\tau}_{3}^{2}$ | $\hat{\tau}_{1}^{2}$ | $\hat{\tau}_{2}^{2}$ | $\hat{\tau}_{3}^{2}$ | $\hat{\rho}_{b12}$ | $\hat{\rho}_{b13}$ | $\hat{\rho}_{b23}$ | $\hat{\rho}_{b12}$ | $\hat{\rho}_{b13}$ | $\hat{\rho}_{b23}$ |
| MV | COM | 25% | -1 | 0 | 0 | 0 | 0 | 0 | 95.6 | 95.7 | 95.6 | 17 | 17 | 17 | -6 | 0 | 0 | -16 | -15 | -17 | 15 | 14.8 | 15.7 |
| UV | COM |  | -1 | 0 | 0 | 0 | 0 | 0 | 95.3 | 95.2 | 95.1 | 6 | 6 | 6 | 0 | 0 | 0 | -- | -- | -- | -- | -- | -- |
| MV | COM^c^ |  | -1 | 0 | 0 | 0 | 0 | 0 | 96.4 | 96.4 | 96.4 | 39 | 50 | 44 | 6 | 12 | 6 | 49 | 74 | 31 | 56.7 | 56.4 | 57.3 |
| MV | MAR |  | -1 | 0 | 0 | 0 | -3 | 0 | 95.6 | 95.4 | 95.6 | 17 | 22 | 17 | -6 | -5 | 0 | -21 | -15 | -21 | 18.3 | 17.9 | 19 |
| UV | MAR |  | -1 | 0 | 0 | 0 | 0 | 0 | 95.3 | 95.3 | 95.1 | 6 | 11 | 6 | 0 | 0 | 0 | -- | -- | -- | -- | -- | -- |
| MV | MIF |  | -1 | 25 | 0 | 0 | -13 | 0 | 95.7 | 64.9 | 95.7 | 17 | -22 | 17 | -6 | -6 | 0 | -32 | -15 | -33 | 25.5 | 24.2 | 25.9 |
| UV | MIF |  | -1 | 31 | 0 | 0 | 0 | 0 | 95.3 | 54.7 | 95.1 | 6 | -39 | 6 | 0 | 0 | 0 | -- | -- | -- | -- | -- | -- |
| MV | COM | 50% | 0 | 0 | 0 | 0 | 0 | 1 | 94.7 | 94.6 | 94.8 | 2 | 2 | 2 | 0 | 0 | 0 | -4 | -1 | -1 | 1.3 | 0.8 | 1.6 |
| UV | COM |  | 0 | 0 | 0 | 0 | 0 | 0 | 94.6 | 94.5 | 94.8 | 0 | 0 | 2 | 0 | 0 | 0 | -- | -- | -- | -- | -- | -- |
| MV | COM^c^ |  | 0 | 0 | 0 | 1 | 0 | 1 | 94.8 | 95.0 | 95.0 | 7 | 9 | 9 | -4 | 0 | 0 | 33 | 48 | 25 | 9.8 | 9 | 11.5 |
| MV | MAR |  | 0 | -1 | 0 | 0 | -4 | 0 | 94.7 | 93.9 | 94.8 | 2 | 4 | 4 | -4 | -3 | 0 | -6 | -2 | -3 | 1.6 | 1 | 2.5 |
| UV | MAR |  | 0 | -1 | 0 | 0 | 0 | 0 | 94.6 | 95.0 | 94.8 | 0 | 0 | 2 | 0 | 0 | 0 | -- | -- | -- | -- | -- | -- |
| MV | MIF |  | 0 | 24 | 0 | 0 | -19 | 1 | 94.7 | 71.7 | 94.8 | 2 | -6 | 4 | 0 | -5 | 0 | -8 | -1 | -5 | 2.2 | 1.4 | 2.7 |
| UV | MIF |  | 0 | 35 | 0 | 0 | 0 | 0 | 94.6 | 62.2 | 94.8 | 0 | -11 | 2 | 0 | 0 | 0 | -- | -- | -- | -- | -- | -- |
| MV | COM | 75% | -1 | -1 | 0 | 0 | 0 | 0 | 95.6 | 95.1 | 95.4 | 0 | 0 | 1 | 0 | 0 | 0 | 0 | -2 | 0 | 0 | 0 | 0.1 |
| UV | COM |  | -1 | -1 | 0 | 0 | 0 | 0 | 95.7 | 94.8 | 95.5 | 0 | 0 | 1 | 0 | 0 | 0 | -- | -- | -- | -- | -- | -- |
| MV | COM^c^ |  | -1 | -1 | 0 | 0 | 0 | 0 | 95.4 | 95.1 | 95.4 | 1 | 1 | 1 | 0 | 0 | 0 | 16 | 18 | 13 | 0.1 | 0 | 0.6 |
| MV | MAR |  | -1 | -1 | 0 | 0 | -6 | 0 | 95.6 | 94.5 | 95.5 | 0 | 1 | 1 | 0 | -2 | 0 | 0 | -2 | 0 | 0.1 | 0 | 0.4 |
| UV | MAR |  | -1 | -1 | 0 | 0 | 0 | 0 | 95.7 | 95.4 | 95.5 | 0 | 0 | 1 | 0 | 0 | 0 | -- | -- | -- | -- | -- | -- |
| MV | MIF |  | 0 | 19 | 0 | 0 | -24 | 0 | 95.6 | 85.2 | 95.3 | 0 | 15 | 1 | 0 | -11 | 0 | 1 | -2 | 1 | 0.2 | 0 | 0.2 |
| UV | MIF |  | -1 | 37 | 0 | 0 | 0 | 0 | 95.7 | 77.7 | 95.5 | 0 | 20 | 1 | 0 | 0 | 0 | -- | -- | -- | -- | -- | -- |

Abbreviation: RMSE, root mean square error; MV, multivariate meta-analysis; UV, Univariate meta-analysis; COM, complete data scenario; MAR, end point 2 missing at random for 30% studies; MAR, end point 2 missing informatively for 30% studies.

^a^The between-study variances for both end-points $\tau_{j}^{2}, j=1,2$ are: $\tau_{j}^{2}$=0.0018 for $I^{2}$=25, $\tau_{j}^{2}$=0.0054 for $I^{2}$=50, $\tau_{j}^{2}$=0.0162 for $I^{2}$=75.

^b^RMSE of estimates by MV method are expressed as % smaller (-) or larger (+) of corresponding estimates by UV method.

^c^$\hat{\rho}_{w}$ignored.

**2. Performance evaluations using directly sampled aggregate data**

**Table G. Relative mean bias percentage, RMSE and coverage probability when**$\mathbf{m=10,}\boldsymbol{\beta}_{\boldsymbol{1}}\boldsymbol{=0.1,}\boldsymbol{\beta}_{\boldsymbol{2}}\boldsymbol{=0.1}$**,** $\boldsymbol{\rho}_{\boldsymbol{b}}$**=0.5,** $\boldsymbol{\rho}_{\boldsymbol{w}}$**=0.5,** $\boldsymbol{s}_{\boldsymbol{j}}^{\boldsymbol{2}}\mathbf{=.0036}$**^a^ (**$\boldsymbol{j=1,2}$**)**

| Method | Scenario | $I^{2}$,^b^ | Effects | | | | | | Heterogeneity | | | | Correlation | | | |
| --- | --- | --- | --- | --- | --- | --- | --- | --- | --- | --- | --- | --- | --- | --- | --- | --- |
|  |  |  | % Bias | | RMSE^c^ | | Coverage Probability | | % Bias | | RMSE^c^ | | % Bias  ($\hat{\rho}_{b}$) | %($\hat{\rho}_{b})$ | | |
|  |  |  | $\hat{\beta}_{1}$ | $\hat{\beta}_{2}$ | $\hat{\beta}_{1}$ | $\hat{\beta}_{2}$ | $\beta_{1}$ | $\beta_{2}$ | $\hat{\tau}_{1}^{2}$ | $\hat{\tau}_{2}^{2}$ | $\hat{\tau}_{1}^{2}$ | $\hat{\tau}_{2}^{2}$ |  | -1 | +1 | \|1\| |
| MV | COM | 25% | 0 | 0 | 0 | 0 | 97.3 | 97.3 | 42 | 42 | 0 | 0 | -31 | 22.6 | 43.7 | 66.4 |
| UV | COM |  | 0 | 0 | 0 | 0 | 97.0 | 96.9 | 25 | 25 | 0 | 0 | -- | -- | -- | -- |
| MV | COM^d^ |  | 0 | 0 | 0 | 0 | 97.7 | 97.8 | 75 | 75 | 10 | 16 | 62 | 3.9 | 78.7 | 82.6 |
| MV | MAR |  | 0 | 0 | 0 | -3 | 97.4 | 97.2 | 42 | 58 | 0 | 0 | -33 | 23.7 | 46.8 | 70.5 |
| UV | MAR |  | 0 | 0 | 0 | 0 | 97.0 | 98.2 | 25 | 42 | 0 | 0 | -- | -- | -- | -- |
| MV | MIF |  | 0 | 20 | 0 | -11 | 97.3 | 89.4 | 42 | -8 | 0 | -5 | -55 | 28.9 | 43.9 | 72.7 |
| UV | MIF |  | 0 | 26 | 0 | 0 | 97.0 | 88.6 | 25 | -25 | 0 | 0 | -- | -- | -- | -- |
| MV | COM | 50% | 0 | 0 | 0 | 0 | 95.3 | 95.2 | 6 | 6 | 0 | -3 | -22 | 13.2 | 26.4 | 39.6 |
| UV | COM |  | 0 | 0 | 0 | 0 | 94.9 | 94.7 | 0 | 0 | 0 | 0 | -- | -- | -- | -- |
| MV | COM^d^ |  | 0 | 0 | 0 | 0 | 95.5 | 95.7 | 11 | 11 | 0 | 0 | 60 | 3.2 | 63.5 | 66.7 |
| MV | MAR |  | 0 | 0 | 0 | -2 | 95.3 | 95.2 | 6 | 8 | 0 | -3 | -22 | 16.2 | 35.5 | 51.6 |
| UV | MAR |  | 0 | 0 | 0 | 0 | 94.9 | 95.9 | 0 | 6 | 0 | 0 | -- | -- | -- | -- |
| MV | MIF |  | 0 | 24 | 0 | -8 | 95.4 | 82.1 | 6 | -17 | 0 | -2 | -41 | 22.7 | 38.5 | 61.2 |
| UV | MIF |  | 0 | 31 | 0 | 0 | 94.9 | 81.6 | 0 | -22 | 0 | 0 | -- | -- | -- | -- |
| MV | COM | 75% | 1 | 1 | 0 | 0 | 95.1 | 94.8 | -1 | 0 | -1 | 0 | -10 | 2.6 | 5.4 | 7.9 |
| UV | COM |  | 1 | 1 | 0 | 0 | 95.0 | 94.8 | -1 | 0 | 0 | 0 | -- | -- | -- | -- |
| MV | COM^d^ |  | 1 | 1 | 0 | 0 | 95.1 | 94.9 | 0 | 2 | -1 | 0 | 31 | 0.9 | 21.1 | 22 |
| MV | MAR |  | 1 | 1 | 0 | -2 | 95.0 | 93.6 | 0 | 6 | -1 | 7 | -12 | 5.5 | 12.9 | 18.4 |
| UV | MAR |  | 1 | 1 | 0 | 0 | 95.0 | 95.3 | -1 | 2 | 0 | 0 | -- | -- | -- | -- |
| MV | MIF |  | 1 | 29 | 0 | -6 | 95.1 | 78.9 | 0 | 6 | -1 | -1 | -18 | 8.5 | 15.2 | 23.7 |
| UV | MIF |  | 1 | 37 | 0 | 0 | 95.0 | 79.6 | -1 | 6 | 0 | 0 | -- | -- | -- | -- |

Abbreviation: RMSE, root mean square error; MV, multivariate meta-analysis; UV, Univariate meta-analysis; COM, complete data scenario; MAR, end point 2 missing at random for $30\%=3$ studies; MAR, end point 2 missing informatively for $30\%=3$ studies.

^a^Corresponds to the IPD meta-analysis scenario of average study size of $n=N/m=1000$ (e.g., for $N=10000$ and $m=10$)

^b^The between-study variances for both end-points $\tau_{j}^{2}, j=1,2$ are: $\tau_{j}^{2}$=0.0012 for $I^{2}$=25%, $\tau_{j}^{2}$=0.0036 for $I^{2}$=50%, $\tau_{j}^{2}$=0.0108 for $I^{2}$=75%.

^c^RMSE of estimates by MV method are expressed as % smaller (-) or % larger (+) of corresponding estimates by UV method.

^d^$\hat{\rho}_{w}$ ignored.

**Table H. Relative mean bias percentage, RMSE and coverage probability when**$\mathbf{m=10,}\boldsymbol{\beta}_{\boldsymbol{1}}\boldsymbol{=0.1,}\boldsymbol{\beta}_{\boldsymbol{2}}\boldsymbol{=0.1}$**,** $\boldsymbol{\rho}_{\boldsymbol{b}}$**=0.75,** $\boldsymbol{\rho}_{\boldsymbol{w}}$**=0.75,** $\boldsymbol{s}_{\boldsymbol{j}}^{\boldsymbol{2}}\mathbf{=.0036}$**^a^ (**$\boldsymbol{j=1,2}$**)**

| Method | Scenario | $I^{2}$,^b^ | Effects | | | | | | Heterogeneity | | | | Correlation | | | |
| --- | --- | --- | --- | --- | --- | --- | --- | --- | --- | --- | --- | --- | --- | --- | --- | --- |
|  |  |  | % Bias | | RMSE^c^ | | Coverage Probability | | % Bias | | RMSE^c^ | | % Bias  ($\hat{\rho}_{b}$) | %($\hat{\rho}_{b})$ | | |
|  |  |  | $\hat{\beta}_{1}$ | $\hat{\beta}_{2}$ | $\hat{\beta}_{1}$ | $\hat{\beta}_{2}$ | $\beta_{1}$ | $\beta_{2}$ | $\hat{\tau}_{1}^{2}$ | $\hat{\tau}_{2}^{2}$ | $\hat{\tau}_{1}^{2}$ | $\hat{\tau}_{2}^{2}$ |  | -1 | +1 | \|1\| |
| MV | COM | 25% | 1 | 0 | 0 | 0 | 97.6 | 97.0 | 42 | 42 | 0 | 0 | -27 | 15.0 | 50.6 | 65.6 |
| UV | COM |  | 1 | 0 | 0 | 0 | 97.2 | 96.6 | 25 | 25 | 0 | 0 | -- | -- | -- | -- |
| MV | COM^d^ |  | 1 | 0 | 0 | 0 | 98.2 | 98.0 | 100 | 108 | 26 | 32 | 27 | 0.1 | 93 | 93.1 |
| MV | MAR |  | 1 | 0 | 0 | -9 | 97.6 | 97.1 | 42 | 50 | 0 | -8 | -29 | 15.3 | 54.9 | 70.2 |
| UV | MAR |  | 1 | 0 | 0 | 0 | 97.2 | 97.9 | 25 | 42 | 0 | 0 | -- | -- | -- | -- |
| MV | MIF |  | 1 | 13 | 0 | -25 | 97.5 | 92.9 | 42 | 0 | 0 | 0 | -42 | 19.7 | 54.2 | 73.9 |
| UV | MIF |  | 1 | 27 | 0 | 0 | 97.2 | 88.2 | 25 | -25 | 0 | 0 | -- | -- | -- | -- |
| MV | COM | 50% | 0 | 1 | 0 | 0 | 96.1 | 96.1 | 8 | 8 | 0 | 0 | -16 | 9.2 | 29.9 | 39.0 |
| UV | COM |  | 0 | 1 | 0 | 0 | 96.0 | 95.7 | 6 | 3 | 0 | 0 | -- | -- | -- | -- |
| MV | COM^d^ |  | 0 | 1 | 0 | 0 | 97.1 | 97.0 | 31 | 28 | 9 | 6 | 30 | 0.3 | 93.8 | 94.1 |
| MV | MAR |  | 0 | 1 | 0 | -8 | 96.2 | 95.8 | 8 | 11 | 0 | -5 | -16 | 10.5 | 41.4 | 51.9 |
| UV | MAR |  | 0 | 1 | 0 | 0 | 96.0 | 96.3 | 6 | 6 | 0 | 0 | -- | -- | -- | -- |
| MV | MIF |  | 0 | 16 | 0 | -22 | 96.3 | 87.9 | 8 | -11 | 0 | -7 | -26 | 15.1 | 44.8 | 59.9 |
| UV | MIF |  | 0 | 32 | 0 | 0 | 96.0 | 81.5 | 6 | -22 | 0 | 0 | -- | -- | -- | -- |
| MV | COM | 75% | 0 | 0 | 0 | 0 | 94.8 | 94.6 | -2 | 0 | -2 | -1 | -7 | 1.7 | 6.7 | 8.4 |
| UV | COM |  | 0 | 0 | 0 | 0 | 94.9 | 94.6 | -2 | 0 | 0 | 0 | -- | -- | -- | -- |
| MV | COM^d^ |  | 0 | 0 | 0 | 0 | 95.4 | 95.2 | 3 | 5 | 0 | 0 | 24 | 0.2 | 61.9 | 62.2 |
| MV | MAR |  | 0 | 0 | 0 | -7 | 94.9 | 93.9 | -2 | 2 | -2 | -2 | -7 | 3.1 | 15.8 | 18.9 |
| UV | MAR |  | 0 | 0 | 0 | 0 | 94.9 | 95.3 | -2 | 0 | 0 | 0 | -- | -- | -- | -- |
| MV | MIF |  | 0 | 16 | 0 | -17 | 95.0 | 83.8 | -1 | 2 | -2 | -13 | -11 | 5.6 | 18.6 | 24.2 |
| UV | MIF |  | 0 | 35 | 0 | 0 | 94.9 | 80.5 | -2 | 6 | 0 | 0 | -- | -- | -- | -- |

Abbreviation: RMSE, root mean square error; MV, multivariate meta-analysis; UV, Univariate meta-analysis; COM, complete data scenario; MAR, end point 2 missing at random for $30\%=3$ studies; MAR, end point 2 missing informatively for $30\%=3$ studies.

^a^Corresponds to the IPD meta-analysis scenario of average study size of $n=N/m=1000$ (e.g., for $N=10000$ and $m=10$)

^b^The between-study variances for both end-points $\tau_{j}^{2}, j=1,2$ are: $\tau_{j}^{2}$=0.0012 for $I^{2}$=25%, $\tau_{j}^{2}$=0.0036 for $I^{2}$=50%, $\tau_{j}^{2}$=0.0108 for $I^{2}$=75%.

^c^RMSE of estimates by MV method are expressed as % smaller (-) or % larger (+) of corresponding estimates by UV method.

^d^$\hat{\rho}_{w}$ ignored.

**Table I. Relative mean bias percentage, RMSE and coverage probability when**$\mathbf{m=15,}\boldsymbol{\beta}_{\boldsymbol{1}}\boldsymbol{=0.1,}\boldsymbol{\beta}_{\boldsymbol{2}}\boldsymbol{=0.1}$**,** $\boldsymbol{\rho}_{\boldsymbol{b}}$**=0.5,** $\boldsymbol{\rho}_{\boldsymbol{w}}$**=0.5,** $\boldsymbol{s}_{\boldsymbol{j}}^{\boldsymbol{2}}\mathbf{=.0025}$**^a^ (**$\boldsymbol{j=1,2}$**)**

| Method | Scenario | $I^{2}$,^b^ | Effects | | | | | | Heterogeneity | | | | Correlation | | | |
| --- | --- | --- | --- | --- | --- | --- | --- | --- | --- | --- | --- | --- | --- | --- | --- | --- |
|  |  |  | % Bias | | RMSE^c^ | | Coverage Probability | | % Bias | | RMSE^c^ | | % Bias  ($\hat{\rho}_{b}$) | %($\hat{\rho}_{b})$ | | |
|  |  |  | $\hat{\beta}_{1}$ | $\hat{\beta}_{2}$ | $\hat{\beta}_{1}$ | $\hat{\beta}_{2}$ | $\beta_{1}$ | $\beta_{2}$ | $\hat{\tau}_{1}^{2}$ | $\hat{\tau}_{2}^{2}$ | $\hat{\tau}_{1}^{2}$ | $\hat{\tau}_{2}^{2}$ |  | -1 | +1 | \|1\| |
| MV | COM | 25% | 0 | 0 | 0 | 0 | 95.5 | 96.6 | 25 | 25 | 0 | 0 | -33 | 20.5 | 39.2 | 59.6 |
| UV | COM |  | 0 | 0 | 0 | 0 | 95.4 | 96.1 | 12 | 12 | 0 | 0 | -- | -- | -- | -- |
| MV | COM^d^ |  | 0 | 0 | 0 | 1 | 96.4 | 97.1 | 62 | 62 | 10 | 20 | 77 | 2.0 | 83.6 | 85.6 |
| MV | MAR |  | 0 | 0 | 0 | -3 | 95.6 | 96.0 | 25 | 38 | 0 | -8 | -34 | 22.3 | 44.7 | 67.0 |
| UV | MAR |  | 0 | 0 | 0 | 0 | 95.4 | 96.7 | 12 | 25 | 0 | 0 | -- | -- | -- | -- |
| MV | MIF |  | 0 | 19 | 0 | -14 | 95.8 | 80.6 | 25 | -50 | 0 | -11 | -64 | 31.6 | 43.9 | 75.5 |
| UV | MIF |  | 0 | 23 | 0 | 0 | 95.4 | 75.8 | 12 | -62 | 0 | 0 | -- | -- | -- | -- |
| MV | COM | 50% | 0 | 0 | 1 | 0 | 94.7 | 95.1 | 4 | 4 | 0 | 0 | -11 | 7.8 | 17.0 | 24.8 |
| UV | COM |  | 0 | 0 | 0 | 0 | 94.9 | 95.1 | 0 | 0 | 0 | 0 | -- | -- | -- | -- |
| MV | COM^d^ |  | 0 | 0 | 1 | 1 | 95.5 | 95.8 | 12 | 12 | 0 | 6 | 69 | 1.1 | 58.2 | 59.4 |
| MV | MAR |  | 0 | 0 | 1 | -3 | 94.9 | 94.3 | 4 | 8 | 0 | 0 | -15 | 11.0 | 26.2 | 37.2 |
| UV | MAR |  | 0 | 0 | 0 | 0 | 94.9 | 95.3 | 0 | 4 | 0 | 0 | -- | -- | -- | -- |
| MV | MIF |  | 0 | 24 | 0 | -12 | 95.0 | 68.8 | 4 | -40 | 0 | -4 | -39 | 21.1 | 34.1 | 55.2 |
| UV | MIF |  | 0 | 30 | 0 | 0 | 94.9 | 64.2 | 0 | -48 | 0 | 0 | -- | -- | -- | -- |
| MV | COM | 75% | 0 | 0 | 0 | 0 | 95.1 | 94.9 | 0 | 0 | 0 | 0 | -3 | 0.5 | 1.0 | 1.5 |
| UV | COM |  | 0 | 0 | 0 | 0 | 95.2 | 95.0 | 0 | 0 | 0 | 0 | -- | -- | -- | -- |
| MV | COM^d^ |  | 0 | 0 | 0 | 0 | 95.2 | 94.9 | 1 | 1 | 0 | 0 | 35 | 0.1 | 10.5 | 10.6 |
| MV | MAR |  | 0 | 0 | 0 | -3 | 95.1 | 93.3 | 0 | 1 | 0 | 4 | -4 | 1.8 | 4.9 | 6.7 |
| UV | MAR |  | 0 | 0 | 0 | 0 | 95.2 | 94.2 | 0 | 0 | 0 | 0 | -- | -- | -- | -- |
| MV | MIF |  | 0 | 30 | 0 | -11 | 95.2 | 69.7 | 0 | -11 | 0 | -2 | -15 | 5.1 | 8.7 | 13.7 |
| UV | MIF |  | 0 | 38 | 0 | 0 | 95.2 | 67.3 | 0 | -11 | 0 | 0 | -- | -- | -- | -- |

Abbreviation: RMSE, root mean square error; MV, multivariate meta-analysis; UV, Univariate meta-analysis; COM, complete data scenario; MAR, end point 2 missing at random for $30\%=5$ studies; MAR, end point 2 missing informatively for $30\%=5$ studies.

^a^Corresponds to the IPD meta-analysis scenario of average study size of $n=N/m=1333$ (e.g., for $N=20000$ and $m=15$)

^b^The between-study variances for both end-points $\tau_{j}^{2}, j=1,2$ are: $\tau_{j}^{2}$=0.0008 for $I^{2}$=25%, $\tau_{j}^{2}$=0.0025 for $I^{2}$=50%, $\tau_{j}^{2}$=0.0075 for $I^{2}$=75%.

^c^RMSE of estimates by MV method are expressed as % smaller (-) or % larger (+) of corresponding estimates by UV method.

^d^$\hat{\rho}_{w}$ ignored.

**Table J. Relative mean bias percentage, RMSE and coverage probability when**$\mathbf{m=15,}\boldsymbol{\beta}_{\boldsymbol{1}}\boldsymbol{=0.1,}\boldsymbol{\beta}_{\boldsymbol{2}}\boldsymbol{=0.1}$**,** $\boldsymbol{\rho}_{\boldsymbol{b}}$**=0.75,** $\boldsymbol{\rho}_{\boldsymbol{w}}$**=0.75,** $\boldsymbol{s}_{\boldsymbol{j}}^{\boldsymbol{2}}\mathbf{=.0025}$**^a^ (**$\boldsymbol{j=1,2}$**)**

| Method | Scenario | $I^{2}$,^b^ | Effects | | | | | | Heterogeneity | | | | Correlation | | | |
| --- | --- | --- | --- | --- | --- | --- | --- | --- | --- | --- | --- | --- | --- | --- | --- | --- |
|  |  |  | % Bias | | RMSE^c^ | | Coverage Probability | | % Bias | | RMSE^c^ | | % Bias  ($\hat{\rho}_{b}$) | %($\hat{\rho}_{b})$ | | |
|  |  |  | $\hat{\beta}_{1}$ | $\hat{\beta}_{2}$ | $\hat{\beta}_{1}$ | $\hat{\beta}_{2}$ | $\beta_{1}$ | $\beta_{2}$ | $\hat{\tau}_{1}^{2}$ | $\hat{\tau}_{2}^{2}$ | $\hat{\tau}_{1}^{2}$ | $\hat{\tau}_{2}^{2}$ |  | -1 | +1 | \|1\| |
| MV | COM | 25% | 0 | 0 | 0 | 0 | 96.4 | 96.6 | 25 | 25 | 0 | 0 | -22 | 12.3 | 46.8 | 59.1 |
| UV | COM |  | 0 | 0 | 0 | 0 | 96.0 | 96.3 | 12 | 12 | 0 | 0 | -- | -- | -- | -- |
| MV | COM^d^ |  | 0 | 0 | 1 | 1 | 97.7 | 97.9 | 100 | 100 | 40 | 40 | 30 | 0.1 | 97.1 | 97.1 |
| MV | MAR |  | 0 | 0 | 0 | -10 | 96.5 | 96.4 | 25 | 38 | 0 | -8 | -25 | 13.3 | 51.3 | 64.7 |
| UV | MAR |  | 0 | 0 | 0 | 0 | 96.0 | 96.5 | 12 | 25 | 0 | 0 | -- | -- | -- | -- |
| MV | MIF |  | 0 | 12 | 0 | -32 | 96.3 | 88.5 | 25 | -25 | 0 | 0 | -40 | 19.5 | 54.4 | 73.9 |
| UV | MIF |  | 0 | 23 | 0 | 0 | 96.0 | 75.2 | 12 | -62 | 0 | 0 | -- | -- | -- | -- |
| MV | COM | 50% | 0 | 0 | 0 | 0 | 94.5 | 94.9 | 0 | 0 | 0 | 0 | -9 | 4.8 | 19.2 | 24 |
| UV | COM |  | 0 | 0 | 0 | 0 | 94.5 | 95.0 | 0 | 0 | 0 | 0 | -- | -- | -- | -- |
| MV | COM^d^ |  | 0 | 0 | 1 | 1 | 95.8 | 96.0 | 24 | 28 | 6 | 6 | 32 | 0 | 94.7 | 94.7 |
| MV | MAR |  | 0 | 0 | 0 | -9 | 94.8 | 94.6 | 0 | 4 | 0 | -5 | -10 | 6.4 | 30.7 | 37.1 |
| UV | MAR |  | 0 | 0 | 0 | 0 | 94.5 | 95.2 | 0 | 0 | 0 | 0 | -- | -- | -- | -- |
| MV | MIF |  | 0 | 16 | 0 | -29 | 94.8 | 79.9 | 4 | -36 | 0 | -5 | -26 | 13.8 | 39.6 | 53.4 |
| UV | MIF |  | 0 | 31 | 0 | 0 | 94.5 | 63.7 | 0 | -48 | 0 | 0 | -- | -- | -- | -- |
| MV | COM | 75% | 0 | 0 | 0 | 0 | 95.1 | 95.4 | -1 | 1 | 0 | 0 | -5 | 0.4 | 1.4 | 1.8 |
| UV | COM |  | 0 | 0 | 0 | 0 | 95.2 | 95.6 | -1 | 1 | 0 | 0 | -- | -- | -- | -- |
| MV | COM^d^ |  | 0 | 0 | 1 | 0 | 95.5 | 95.7 | 3 | 5 | 0 | 0 | 24 | 0.1 | 48.9 | 48.9 |
| MV | MAR |  | 0 | 0 | 0 | -8 | 95.1 | 93.9 | -1 | 1 | 0 | -2 | -6 | 1.2 | 6 | 7.2 |
| UV | MAR |  | 0 | 0 | 0 | 0 | 95.2 | 95.1 | -1 | 1 | 0 | 0 | -- | -- | -- | -- |
| MV | MIF |  | 0 | 19 | 0 | -25 | 95.3 | 77.2 | -1 | -9 | 0 | -7 | -12 | 3.5 | 9.4 | 12.9 |
| UV | MIF |  | 0 | 39 | 0 | 0 | 95.2 | 68.2 | -1 | -9 | 0 | 0 | -- | -- | -- | -- |

Abbreviation: RMSE, root mean square error; MV, multivariate meta-analysis; UV, Univariate meta-analysis; COM, complete data scenario; MAR, end point 2 missing at random for $30\%=5$ studies; MAR, end point 2 missing informatively for $30\%=5$ studies.

^a^Corresponds to the IPD meta-analysis scenario of average study size of $n=N/m=1333$ (e.g., for $N=20000$ and $m=15$)

^b^The between-study variances for both end-points $\tau_{j}^{2}, j=1,2$ are: $\tau_{j}^{2}$=0.0008 for $I^{2}$=25%, $\tau_{j}^{2}$=0.0025 for $I^{2}$=50%, $\tau_{j}^{2}$=0.0075 for $I^{2}$=75%.

^c^RMSE of estimates by MV method are expressed as % smaller (-) or % larger (+) of corresponding estimates by UV method.

^d^$\hat{\rho}_{w}$ ignored.
